# *Leptospira* Species Infection and Seropositivity in Domestic Livestock and Feral Swine in Puerto Rico

**DOI:** 10.1155/tbed/2538118

**Published:** 2026-05-27

**Authors:** Katherine L. Dirsmith, Jarlath E. Nally, Graham Sutherland, Camila Hamond, Renee L. Galloway, Karen LeCount, Tammy Anderson, Fred V. Soltero, Saraí Rivera-García, Victoria Novak, Gustavo E. Olivieri-Cintrón, Kenneth Gruver, Vienna R. Brown, Clayton Glassey, A. Shane McKinley, Oniel Candelaria, Leif Stephens, Ilana J. Schafer

**Affiliations:** ^1^ Animal and Plant Health Inspection Service, U.S. Department of Agriculture, Veterinary Services Field Operations, San Juan, Puerto Rico, 00918, USA, usda.gov; ^2^ National Center for Animal Health Leptospira Working Group, U.S. Department of Agriculture, Ames, Iowa, 50010, USA, usda.gov; ^3^ Infectious Bacterial Diseases Research Unit, Agricultural Research Service, U.S. Department of Agriculture, Ames, Iowa, 50010, USA, usda.gov; ^4^ Bacterial Special Pathogens Branch, Division of High-Consequence Pathogens and Pathology, Centers for Disease Control and Prevention, Atlanta, Georgia, 30329, USA, cdc.gov; ^5^ National Veterinary Services Laboratories, Animal and Plant Health Inspection Service, U.S. Department of Agriculture, Ames, Iowa, 50010, USA, usda.gov; ^6^ Animal and Plant Health Inspection Service, U.S. Department of Agriculture, Wildlife Services, Alabama/Puerto Rico/USVI Program, 6155 Heath Road, Auburn, Alabama, 36830, USA, usda.gov; ^7^ Animal and Plant Health Inspection Service, U.S. Department of Agriculture, Wildlife Services, National Feral Swine Damage Management Program, Fort Collins, Colorado, 80521, USA, usda.gov

## Abstract

Leptospirosis is a zoonotic disease of global importance that significantly impacts human health and the livestock industry. In Puerto Rico, outbreaks of leptospirosis in humans have been associated with hurricanes and subsequent flooding events. Rodent species in Puerto Rico and feral swine in the continental United States have been implicated as reservoir hosts for *Leptospira* species and serogroups, with the possibility of transmitting the pathogen to humans. In this cross‐sectional investigation, we collected samples from 670 animals, including 375 feral swine, 168 transitional swine, 120 dairy cattle, six beef cattle, and one small ruminant to assess *Leptospira* spp. exposure and infection in specific regions of Puerto Rico. Of all animals sampled and tested, 55.82% (*n* = 374) had a positive result to at least one of the following leptospirosis tests performed: microscopic agglutination test (MAT), polymerase chain reaction (PCR), fluorescent antibody test (FAT), and/or culture. Of all animals from which serum samples were tested, 75.90% (*n* = 126) of transitional swine, 78.72% (*n* = 37) of dairy cattle, 100% (*n* = 4) of beef cattle, and 53.13% (*n* = 195) of feral swine had detectable *Leptospira* antibodies on MAT. Of all animals from which urine samples were collected and tested on either *lipL32* PCR or FAT, 0% of transitional swine, 22.86% (*n* = 24) of dairy cattle, 16.67% (*n* = 1) of beef cattle, and 1.71% (*n* = 2) of feral swine tested positive for *Leptospira* species on one or both of these tests. *L. borgpetersenii* serogroup Sejroe serovar Hardjo was isolated from the urine of two dairy cows, and *L. santarosai* serogroup Pyrogenes was isolated from the urine of another dairy cow. The findings of this investigation provide valuable information on the circulating *Leptospira* species, serogroups, and serovars in domestic livestock and feral swine in specific regions of Puerto Rico and will further inform human and animal disease prevention, preparedness, and response.

## 1. Introduction

*Leptospira* species and serogroups are a large family of aerobic, spirochete bacteria that can live in the environment and are found worldwide. Within the *Leptospira* genus, there are over 70 species, consisting of pathogenic and nonpathogenic species [1–3]. *Leptospira* bacteria are also classified into serovars that differ according to their agglutination patterns with reference antisera targeting surface‐exposed lipopolysaccharide [1]. Over 300 different *Leptospira* serovars have been identified to date and closely related serovars are grouped into serogroups [4, 5]. Paradoxically, the same serogroup can belong to multiple species.

Leptospirosis has been identified as a zoonosis of global public health importance. *Leptospira* spp. and serogroups have been detected in humans and in many other mammalian species worldwide [6]. Annually, over one million leptospirosis cases are estimated to occur in humans, of which approximately 5.7% are fatal [7]. Human patients with leptospirosis in Puerto Rico have been found to commonly have antibodies specific for the serogroups found in rodents, dogs, pigs, and horses [8]. However, serology does not definitively identify infecting serogroups.

A wide variety of domestic animals are affected by *Leptospira* spp. In animals with subclinical infections, a chronic carrier status can occur, particularly in cattle [9], swine [9], sheep [9–13], horses [9, 14], and goats [15–17]. *Leptospira* spp. may persist within these hosts for long periods of time and can be shed into the environment in urine for months to years [9, 18–20]. In the environment, *Leptospira* spp. may remain viable for several months under ideal conditions [5, 21].

Several species of wild animals serve as reservoir hosts of *Leptospira* spp. [5, 6], typically asymptomatic carriers that excrete leptospires [22–24], which may contaminate soil and water [6], including in areas frequented by livestock. A recent study revealed that house mice (*Mus musculus*), black rats (*Rattus rattus*), Norway rats (*Rattus norvegicus*), and small Indian mongooses (*Urva auropunctata*) are important reservoirs of *L. interrogans* in Puerto Rico on cattle farms [25]. Another study demonstrated that 11 of 18 rats (61%) trapped in the Caño Martín Peña community in San Juan, Puerto Rico, carried pathogenic *Leptospira* species in the kidneys, with *L. borgpetersenii* isolated from the urine of one house mouse [26]. Feral swine have also been implicated as reservoir hosts of *Leptospira* spp. in the continental United States [27].

Increased *Leptospira* spp. prevalence has been detected in dense urban and peri‐urban areas with insufficient waste collection and sanitation [28] and poor housing conditions [29–31]. Leptospirosis can also be an occupational disease, which can affect workers whose job requires them to spend time in environments with increased risk of exposure to *Leptospira* spp., including workers in agriculture [32–39], veterinary medicine [40], abattoirs [40–43], sanitation [34, 44], and disaster response [45].

As in other parts of the world [28, 46–52], leptospirosis outbreaks in Puerto Rico are often associated with heavy rains and flooding events. During flooding events, contaminated soil and water can be washed into water bodies or agricultural fields [6]. In these flooded fields, mammals can continue to urinate and shed leptospires into the environment. In several studies, it has been demonstrated that detectable levels of *Leptospira* spp. and serogroups, including those that are pathogenic, are present in a significant percentage of soil and water samples collected throughout Puerto Rico [53, 54].

Several investigations have focused on leptospirosis outbreaks associated with flooding effects caused by hurricanes in Puerto Rico. In the aftermath of Hurricanes Irma and Maria, the largest post‐hurricane leptospirosis outbreak at the time occurred in Puerto Rico (I. Schafer, manuscript in preparation). The case fatality rate was notably large, likely in part due to a greater number of severe cases reported than mild cases. After Hurricane Fiona in 2022, 115 confirmed and probable human cases of leptospirosis were reported [55]. The actual incidence rate of leptospirosis during these times was likely much higher, but not recorded, due to infected, mildly‐clinical to subclinical patients not seeking medical treatment, as well as underdiagnosis and underreporting due to the similarity in clinical signs between leptospirosis and Dengue fever, which is common in Puerto Rico [56].

The urban community of Caño Martín Peña in San Juan, Puerto Rico, lies on the banks of a canal prone to flooding events during significant rainfall. This community has a high population density [57, 58] and in parts, a lack of robust sewage and storm drainage infrastructure [57]. A study revealed that 55 out of 202 (27.2%) human study participants residing in Caño Martín Peña had detectable *Leptospira* antibodies. The distance of a household from the canal was inversely correlated with the detection of antibodies against *Leptospira* serogroups in members of that household [26]. Furthermore, a study detected pathogenic *Leptospira* spp. and serogroups in the majority of soil samples collected at three different points in time along the edge of the flood‐prone Caño Martín Peña canal [54]. A new species of pathogenic *Leptospira*, classified as *Leptospira sanjuanensis*, representing a novel serogroup, was isolated from one of these soil samples [59].

Following Hurricanes Irma and Maria, a more thorough understanding of the prevalence and distribution of *Leptospira* spp. infection and seropositivity in wild and domestic animals in Puerto Rico was needed. Samples for leptospirosis testing were collected opportunistically from feral swine and domestic animals, including swine, dairy and beef cattle, and a small ruminant at locations throughout Puerto Rico. This investigation aimed to (1) detect *Leptospira* spp. infection in domestic livestock and feral swine in specific regions of Puerto Rico, (2) characterize the distribution and prevalence of antibodies against *Leptospira* serogroups, (3) identify circulating *Leptospira* species and serogroups, (4) elucidate animal reservoirs of importance for leptospirosis in Puerto Rico, and (5) inform both human and animal leptospirosis prevention and response efforts.

## 2. Methods

### 2.1. Domestic Livestock Sampling Methodology

From September 2019 to September 2021, opportunistic samples for *Leptospira* spp. testing were collected from domestic livestock, including swine, dairy cattle, beef cattle, and one ewe, in conjunction with United States Department of Agriculture (USDA) Animal and Plant Health Inspection Service (APHIS) Veterinary Services (VS) routine program activities. Samples were obtained upon voluntary consent from the owners. A visual evaluation of the animal was performed prior to sampling. If clinical signs consistent with leptospirosis were noted, a full physical examination was performed by a veterinarian. The selection of animals for full physical examinations was based on clinical signs alone, rather than a specific case definition. Due to frequent subclinical infections reported in domestic animals, leptospirosis suspicion was not ruled out by the absence of clinical signs. Samples collected included serum and urine from healthy animals, in addition to serum and whole blood from sick and euthanized animals, and kidneys from euthanized animals. All euthanized domestic livestock that were sampled in this study were humanely euthanized with the consent of the owners for medical reasons unrelated to this study, as part of routine VS sick animal investigations.

#### 2.1.1. Transitional Swine Samples

In Puerto Rico, the majority of swine farms are small operations classified as “backyard” swine farms, in which the producers own a relatively small number of production animals. The swine on these farms are classified as “transitional” swine because of their increased probability of contact and breeding with feral swine due to farm structure. Serum from transitional swine was collected by VS for leptospirosis testing in conjunction with routine surveillance for other diseases.

Two transitional swine (TSP‐0276 and TSP‐0350) exhibiting clinical signs of disease were euthanized and necropsied at two different backyard swine farms. Kidney samples were collected from both animals, and a whole blood sample was collected from TSP‐0350.

#### 2.1.2. Cattle Samples

VS personnel in Puerto Rico conducted an approximately year‐long cattle tick treatment program at various dairy and beef cattle farms in Puerto Rico, with tick treatment occurring at regular intervals. Serum for leptospirosis testing was collected from randomly‐selected cattle without clinical signs at five farms in conjunction with routine serum sample collection for VS program purposes. Additionally, free‐catch urine samples were collected from randomly‐selected cattle for leptospirosis testing. Mid‐stream urine was prioritized in sample collection to minimize contaminants. Urine for culture was collected into conical tubes with HAN transport media as previously described [60] from five cattle from two different farms based on *lipL32* polymerase chain reaction (PCR) results.

One cow (DCP‐001) with clinical signs of disease was sampled at a sixth farm. A kidney sample was opportunistically collected for *Leptospira* spp. testing.

#### 2.1.3. Small Ruminant Samples

A Katahdin ewe that presented with clinical signs of disease was humanely euthanized and necropsied. Serum and urine samples were opportunistically collected for leptospirosis testing.

### 2.2. Feral Swine Sampling Methodology

From August 12, 2019 to August 16, 2019, over 518 feral swine were humanely trapped by USDA APHIS Wildlife Services (WS) personnel in the Caño Martín Peña neighborhood of San Juan, Puerto Rico using baited traps. Feral swine were transported to an off‐site farm owned by the Puerto Rico Department of Agriculture, where they were placed into holding pens and humanely euthanized by WS personnel as part of damage management protocols, as outlined in WS Program Directive 2.505. WS personnel trapped an additional 393 feral swine from the municipalities of San Juan, Carolina, and Guaynabo on multiple capture days in 2020 and 2021.

USDA APHIS VS personnel collected post‐mortem biological specimens from the swine, including serum, kidneys, and urine. Due to the large number of feral swine euthanized each day and limited personnel, samples for leptospirosis testing could not be collected from all swine, nor could all specimen types be collected from those animals sampled. From two pregnant sows, placental samples, along with post‐mortem fetal serum and kidney samples, were collected.

Serum samples were collected by post‐mortem intracardiac puncture. To collect post‐mortem kidney and urine samples, a ventral midline incision was made on each animal and a sterile urine sample was collected from the bladder with a needle and syringe. One kidney was removed with the capsule intact. Two tubes of culture media, one containing 5 mL of HAN media [61] and the other containing 5 mL of T80/40/LH media [62] supplemented with 100 μg 5‐FU, were inoculated with 2–3 drops of urine each.

For kidney sample processing, the capsule was removed using disinfected forceps. Then, a 2–3 cm wedge‐shaped incision was made in the kidney using a sterile scalpel, extending through the cortex and medulla, to collect an approximately 15–20 g sample of kidney.

### 2.3. Sample Processing and Shipment

Blood samples for serum were placed into serum separator tubes and centrifuged on‐site. Serum was decanted into cryovials. Whole blood samples were placed into EDTA tubes. Urine samples for molecular testing were placed into conical tubes. Kidney samples were placed in sterile polyethylene bags. Serum, whole blood, kidney, and non‐culture urine samples were refrigerated until shipment. They were sent to the laboratory in batches in insulated shipping boxes with ice. Culture media inoculated with urine was stored at room temperature in the dark and shipped at room temperature to the laboratory on the sample collection day. All samples were either shipped to the USDA National Centers for Animal Health (NCAH) *Leptospira* Working Group in Ames, Iowa, or to the Centers for Disease Control and Prevention (CDC) Zoonoses and Select Agent Laboratory (ZSAL) in Atlanta, Georgia.

### 2.4. Data Collection and Analysis

Data collected for each animal included sampling or capture location, animal identification number, age, sex, breed, historical and current clinical signs, and leptospirosis vaccination history (for domestic animals only). All data was collated into a Microsoft Excel database. Transitional swine were considered adult if they were at least 6 months old based on the average age at sexual maturity of both boars and sows [63]. All transitional swine less than 6 months of age were classified as juvenile. Feral swine were classified as adults if they were reproductively active and were classified as subadults if they were no longer nursing but not yet reproductively active. Feral swine were classified as juveniles if they were nursing.

All data analyses and graphs were made using R [64], R Studio [65], and the tidyverse [66] and ggplot [67] packages.

### 2.5. Laboratory Testing

Serum samples were tested with the microscopic agglutination test (MAT) [68] according to WOAH guidelines at an initial dilution of 1:100 against either Panel A (a panel of 19 *Leptospira* serovars representing 16 serogroups at the NCAH) or panel B (a panel of 20 *Leptospira* serovars representing 17 serogroups at the ZSAL) (Supporting information 1: Table S1). Serum samples from all domestic animals, as well as feral swine sampled after 2019, were tested by MAT Panel A. Serum samples from all feral swine sampled in 2019 were tested by MAT Panel B. A MAT titer of ≥1:100 was considered positive.

Urine was processed for the fluorescent antibody test (FAT) as previously described [60] at the NCAH using a rabbit origin multivalent fluorescent antibody conjugate (FITC‐bound).

DNA was extracted from urine, kidney, and whole blood samples and was tested by *lipL32* PCR as previously described [60, 69, 70] at the ZSAL for samples collected in August 2019 and at the NCAH for samples collected from September 2019 to September 2021. Samples were considered positive with cycle threshold (Ct) values <40. For feral swine sampled in August 2019, only urine samples that were positive on FAT were tested on *lipL32* PCR. The only additional feral swine urine samples tested on *lipL32* PCR were those urine samples collected on March 4, 2021.

For selected samples that were positive on *lipL32* PCR, genotyping was performed at the NCAH using *secY* primers as previously described [71–73]. Out of the dairy cattle that yielded positive urine samples on *lipL32* PCR, five cattle with relatively low PCR Ct values from two different farms were selected for urine culture sample collection. Bovine and feral swine urine was cultured and isolates were serotyped at the NCAH laboratory as previously described [60, 74, 75].

## 3. Ethical Approvals

All specimens collected opportunistically from live and euthanized animals that were already being collected by USDA VS or were collected from animals euthanized by USDA WS were considered IACUC exempt by the CDC. Specimens that were collected from live animals specifically for this project were approved under the CDC IACUC protocol #2879SALMULX‐A3.

## 4. Results

Samples were collected and tested from a total of 670 animals (295 domestic animals and 375 feral swine) distributed across Puerto Rico. A total of 374 animals (55.82%) had a positive result on at least one leptospirosis test performed (MAT, PCR, FAT, and/or culture). Positivity by animal group is included in Table 1. Full results of these leptospirosis tests are available in Supporting Information 2: File S1 and Supporting information 3: File S2.

**Table 1:** Number of animals (symptomatic and asymptomatic), sampled in Puerto Rico from August 2019 to September 2021, with positive *Leptospira* spp. results on MAT, *lipL32* PCR, *secY* PCR, FAT, and/or culture.

Animal group	# Animals tested on any test	# Animals positive on at least one test	Serology (percent seropositive)	*lipL32* PCR, urine (percent positive)	*secY* PCR, urine (percent positive)	FAT, urine (percent positive)	Culture, urine (percent positive)
All domestic animals	295	179/295 (60.68%)	167/218 (76.61%)	16/102 (15.69%)	11/14 (78.57%)	8/102 (7.84%)	3/5 (60%)
Transitional swine	168	127/168 (75.60%)	126/166 (75.90%)	0/1 (0%)	N A	0/1 (0%)	N A
Dairy cattle	120	47/120 (39.17%)	37/47 (78.72%)	15/94 (15.96%)	11/14 (78.57%)	8/94 (8.51%)	3/5 (60%)
Beef cattle	6	5/6 (83.33%)	4/4 (100%)	1/6 (16.67%)	N A	0/6 (0%)	N A
Small ruminants	1	0/1 (0%)	0/1 (0%)	0/1 (0%)	N A	0/1 (0%)	N A
Feral swine	375	195/375 (52%)	195/367 (53.13%)	0/8 (0%)	N A	2/117 (1.71%)	0/96 (0%)
All animals	670	374/670 (55.82%)	362/585 (61.88%)	16/110 (14.55%)	11/14 (78.57%)	10/219 (4.57%)	3/101 (2.97%)

*Note*: The denominators indicate the total number of animals tested using the indicated assay. Genotyping was performed using *secY* primers only for selected samples that were positive on *lipL32* PCR. Of the dairy cattle that yielded urine samples positive on *lipL32* PCR, five cattle with relatively early PCR cycle threshold values from two different farms were selected for urine culture sample collection.

### 4.1. Summary

**Figure 1:**
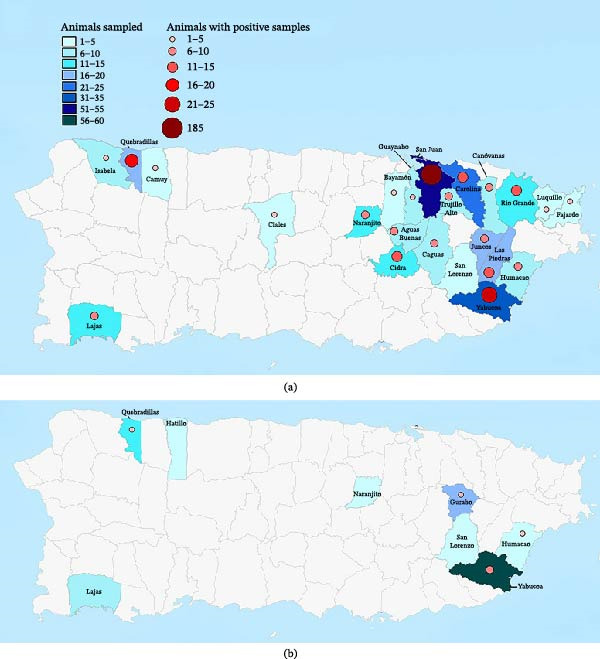
(a) Number of domestic animals and feral swine from which serum samples collected from August 2019 to September 2021 were positive for antibodies against *Leptospira* serogroups, by Puerto Rico municipality. Of 577 animals from which serum samples were collected and municipality data is available, 357 (61.87%) had serum samples with *Leptospira* antibodies. (b) Number of domestic animals and feral swine from which urine and/or kidney samples were positive for *Leptospira* spp. on *lipL32* PCR, by Puerto Rico municipality. Of all 104 animals from which urine and/or kidney samples were collected, 17 (16.35%) animals had at least one sample that was positive on *lipL32* PCR for *Leptospira* spp. *Source*: Esri, CGIAR, USGS (https://services.arcgisonline.com/arcgis/rest/services/Elevation/World_Hillshade/MapServer); Esri, TomTom, Garmin, Foursquare, SafeGraph, FAO, METI/NASA, USGS, NPS, USFWS (https://basemaps.arcgis.com/arcgis/rest/services/World_Basemap_v2/VectorTileServer); Puerto Rico Planning Board; FEMA (https://services.arcgis.com/XG15cJAlne2vxtgt/arcgis/rest/services/Municipios/FeatureServer/0). Image finalized in Adobe Inc., 2019. Adobe Photoshop (https://www.adobe.com/products/photoshop.html).

From September 2019 to September 2021, samples from a total of 295 domestic animals were collected and tested from 54 farms across 25 municipalities in Puerto Rico (Figure 1). Of all domestic animals sampled, 56.95% (*n* = 168) were transitional swine, 40.68% (*n* = 120) were dairy cattle, 2.03% (*n* = 6) were beef cattle, and 0.34% (*n* = 1) were sheep. The transitional swine samples were collected from 46 backyard farms, dairy cattle samples from six farms, and beef cattle samples from two farms. Age, sex, and health status at the time of sampling are summarized in Table 2. All domestic animals sampled and tested were either not vaccinated for leptospirosis (55.25%, *n* = 163) according to the owner, or the vaccination status was unknown (44.75%, *n* = 132).

**Table 2:** Summary of sex, age, and health status of domestic animals and feral swine sampled for *Leptospira* spp. testing in Puerto Rico from August 2019 to September 2021.

Animal group	Sex^a^		Age^b^			Health status		
	Female	Male	Juvenile	Sub‐adult	Adult	Alive (healthy)	Euthanized (sick^c^)	Euthanized (healthy)
All domestic animals	191/232 (82.33%)	41/232 (17.67%)	39/230 (16.96%)	N A	191/230 (83.04%)	291/295 (98.64%)	4/295 (1.36%)	0/295 (0%)
Transitional swine	67/105 (63.81%)	38/105 (36.19%)	39/103 (37.86%)	N A	64/103 (62.14%)	166/168 (98.81%)	2/168 (1.19%)	0/168 (0%)
Dairy cattle	117/120 (97.5%)	3/120 (2.5%)	0/120 (0%)	N A	120/120 (100%)	119/120 (99.17%)	1/120 (0.83%)	0/120 (0%)
Beef cattle	6/6 (100%)	0/6 (0%)	0/6 (0%)	N A	6/6 (100%)	6/6 (100%)	0/6 (0%)	0/6 (0%)
Small ruminants	1/1 (100%)	0/1 (0%)	0/1 (0%)	N A	1/1 (100%)	0/1 (0%)	1/1 (100%)	0/1 (0%)
Feral swine	164/371 (44.20%)	207/371 (55.80%)	25/375 (6.67%)	64/375 (17.07%)	286/375 (76.27%)	0/375 (0%)	0/375 (0%)	375/375 (100%)
All animals	355/603 (58.87%)	248/603 (41.13%)	64/605 (10.58%)	64/375 (17.07%)	477/605 (78.84%)	291/670 (43.43%)	4/670 (0.60%)	375/670 (55.97%)

^a^Percentages represent the number of individuals with the indicated sex out of all individuals in the indicated animal group with a recorded sex status. Sex status was not reported for 63 transitional swine and four feral swine.

^b^Percentages represent the number of individuals with the indicated age status out of all individuals in the indicated animal group with a recorded age status. Age status was not reported for 65 of the transitional swine sampled.

^c^An animal was classified as “sick” if any apparent clinical signs were present upon sampling. In the absence of clinical signs, the animal was classified as “healthy.”

Of the 295 domestic animals sampled and tested, 60.68% (*n* = 179) had a positive result on at least one assay testing for *Leptospira* spp. (Table 1). Upon sampling, 98.64% (*n* = 291) of the domestic animals exhibited no clinical signs of disease and 1.36% (*n* = 4) exhibited clinical signs of disease. Of the sick animals sampled, which were all from different farms: one transitional swine juvenile (TSP‐0276) exhibited clinical signs of prolonged vomiting and diarrhea; one transitional sow (TSP‐0350) exhibited prolonged lateral recumbency, hypothermia, dyspnea, and poor body condition; one cow (DCP‐001) presented with severe lethargy and peripheral edema; and one Katahdin ewe (SRP‐0001) presented with persistent weakness and low body condition score. Of all four symptomatic domestic animals, only the adult transitional sow (TSP‐0350) yielded positive results—the kidney sample was positive for *Leptospira* spp. on *lipL32* PCR, but was negative on FAT and *secY* PCR, and the whole blood was negative on *lipL32* PCR.

From August 2019 to June 2021, samples from a total of 375 feral swine were collected and tested. These feral swine had home ranges in the municipalities of Carolina (*n* = 17), Guaynabo (*n* = 4), and San Juan (*n* = 346). Of the feral swine sampled from San Juan, all were captured in the Caño Martín Peña region. For eight feral swine, capture location was not recorded. Age, sex, and health status at the time of sampling are described in Table 2. Because of their feral status, health history was unknown.

Of the 375 feral swine sampled and tested, 52% (*n* = 195) had a positive result on at least one assay testing for *Leptospira* spp. (Table 1).

### 4.2. Serology

Serum samples from 218 domestic animals were tested by MAT Panel A. *Leptospira* antibodies were detected in 76.61% (*n* = 167) of these animals (Table 1). *Leptospira* antibodies were detected in cattle from all five cattle farms sampled for MAT testing.

Serum samples from 367 feral swine were tested by MAT. *Leptospira* antibodies were detected in 53.13% (*n* = 195) of these animals (Table 1). All feral swine with any positive leptospirosis result on other diagnostic assays were also MAT‐positive. Two of the feral sows captured and humanely euthanized were carrying fetuses. Post‐mortem serum from a fetus from each feral sow was collected and tested by MAT. Both samples were negative for *Leptospira* antibodies. It is important to note that, in the case of serogroups Hebdomadis, Icterohaemorrhagiae, Mini, Pyrogenes, and Sejroe, the specific serovars included on MAT Panel A (used for feral swine serum collected after 2019) differ from those on MAT Panel B (used for feral swine serum collected in 2019). Therefore, serovar‐specific results for these serogroups are not summarized for feral swine. Furthermore, serogroups Celledoni and Javanica are included on MAT Panel B but not on MAT Panel A. Therefore, these serogroups are not included in this analysis. Antibodies against serogroup Celledoni (1:1600) were only detected in one feral swine and antibodies against serogroup Javanica (1:100) were only detected in another feral swine.

**Figure 2:**
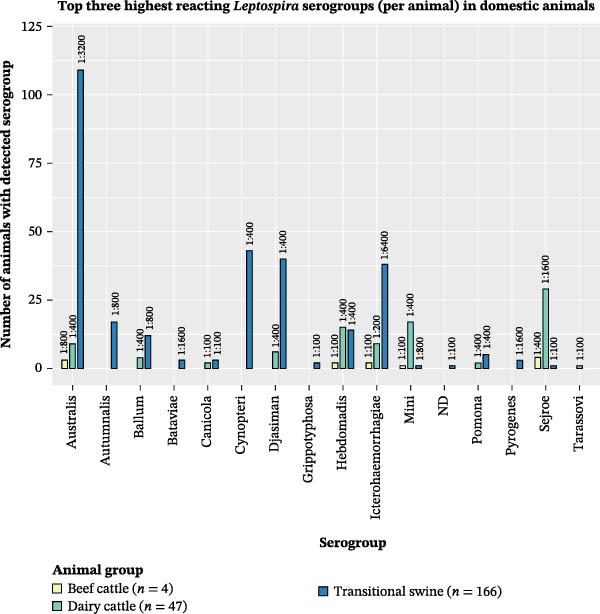
Number of domestic animals sampled in Puerto Rico from September 2019 to September 2021 in which the specified *Leptospira* serogroup was among the top three highest reacting serogroups in an individual sample. The number above each bar represents the highest MAT titer detected of the specified serogroup in the indicated animal group. The numbers in the legend refer to the number of animals in each specific animal group from which serum samples were collected and tested.

**Figure 3:**
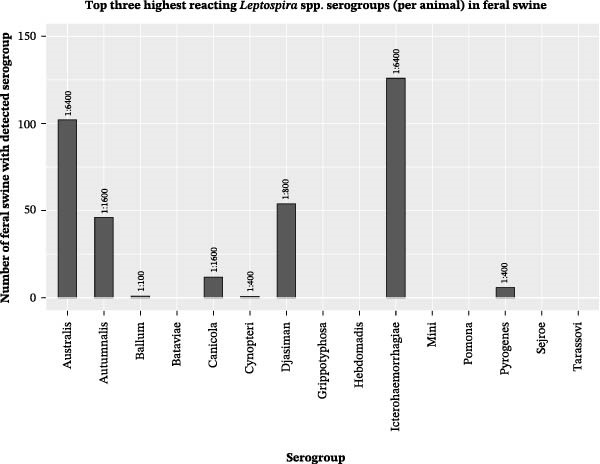
The number of feral swine sampled in Puerto Rico from August 2019 to June 2021 in which the specified *Leptospira* serogroup was among the top three highest reacting serogroups in an individual serum sample. The number above each bar represents the highest MAT titer of all detections of the specified serogroup.

The *Leptospira* serogroups that were most commonly among the top three highest reacting serogroups in individual animals were determined. To do so, the three serogroups with the highest titers on MAT were identified for each animal. Then, the number of animals for which each serogroup was identified among the top three highest reacting was determined (Figures 2 and 3). Table 3 demonstrates the serogroups found most commonly among the top three highest reacting for individuals in each animal group. The top three highest reacting serogroups for each domestic animal group were also the three most frequently reacting serogroups for that animal group, regardless of titer level. The top three highest reacting serogroups in feral swine captured in the Caño Martín Peña region were the same as those in feral swine captured in other regions. These serogroups were also the three most frequently detected serogroups in feral swine, regardless of titer.

**Table 3:** Proportion of animals sampled in Puerto Rico from August 2019 to September 2021 in which the indicated *Leptospira* serogroup was most frequently among the top three highest reacting serogroups on MAT in individuals in the indicated animal group.

Animal group	Most frequently detected highest reacting serogroup	2^nd^ most frequently detected highest reacting serogroup	3^rd^ most frequently detected highest reacting serogroup(s)
Transitional swine	Australis Serovar Bratislava Seropositive = 108/166 (65.06%) Mean = 1:287 Median = 1:200 Range = 1:100–1:3200	Cynopteri Serovar Cynopteri Seropositive = 43/166 (25.90%) Mean = 1:130 Median = 1:100 Range = 1:100–1:400	Djasiman Serovar Djasiman Seropositive = 40/166 (24.10%) Mean = 1:128 Median = 1:100 Range = 1:100–1:400
Dairy cattle	Sejroe^a^ Serovars Sejroe, Hardjo, Recreo Seropositive = 29/47 (61.70%) Mean = 1:351 Median = 1:200 Range = 1:100–1:1600	Mini Serovar Szwajizak Seropositive = 17/47 (36.17%) Mean = 1:152 Median = 1:100 Range = 1:100–1:400	Hebdomadis Serovar Hebdomadis Seropositive = 15/47 (31.91%) Mean = 1:160 Median = 1:100 Range = 1:100–1:400
Beef cattle	Sejroe^b^ Serovars Sejroe and Hardjo Seropositive = 4/4 (100%) Mean = 1:263 Median = 1:200 Range = 1:100–1:400	Australis Serovar Bratislava Seropositive = 3/4 (75%) Mean = 1 : 467 Median = 1 : 400 Range = 1:200–1:800	Hebdomadis and Icterohaemorrhagiae^c^ Serovars Hebdomadis and Copenhageni Seropositive = 2/4 (50%)^c^ Mean = 1:100 Median = 1:100 Range = NA
Small ruminants	NA	NA	NA
Feral swine	Icterohaemorrhagiae Serovars Icterohaemorrhagiae and Mankarso Seropositive = 126/367 (34.33%) Mean = 1:353 Median = 1:200 Range = 1:100–1:6400	Australis^d^ Serovars Australis and Bratislava Seropositive = 105/367 (28.61%) Mean = 1:298 Median = 1:200 Range = 1:100–1:6400	Djasiman Serovar Djasiman Seropositive = 54/367 (14.71%) Mean = 1:183 Median = 1:150 Range = 1:100–1:800

*Note*: The specific *Leptospira* serovars for each serogroup detected by antibody agglutination on MAT is included. The denominators indicate the total number of animals in the indicated animal group with serum samples that were tested by MAT. The mean, median, and range values were calculated for titers when the indicated serogroup was among the top three highest reacting serogroups for the indicated animal group. In some individual animals, antibodies against multiple *Leptospira* spp. serovars within the same serogroup were detected.

^a^Of all detections of antibodies against *Leptospira* serogroup Sejroe in dairy cattle, there were 27 detections of antibodies against serovar Sejroe, 20 detections of antibodies against serovar Hardjo, and five detections of antibodies against serovar Recreo.

^b^Of all detections of antibodies against *Leptospira* serogroup Sejroe in beef cattle, there were four detections of antibodies against serovar Sejroe and four detections of antibodies against serovar Hardjo.

^c^50% of all beef cattle with serum tested on MAT were seropositive for serogroup Hebdomadis, and 50% of all beef cattle with serum tested on MAT were seropositive for serogroup Icterohaemorrhagiae.

^d^Of all detections of antibodies against *Leptospira* serogroup Australis in feral swine, there were 105 detections of antibodies against serovar Bratislava and one detection of antibodies against serovar Australis.

### 4.3. *lipL32* PCR and FAT

Urine was tested by *lipL32* PCR and FAT for *Leptospira* spp. from 102 domestic animals, including 94 dairy cattle, six beef cattle, one transitional swine, and one small ruminant (Table 1). Out of these 102 animals, 15.69% (*n* = 16) tested positive for *Leptospira* spp. by *lipL32* PCR (15.96% of dairy cattle, 16.67% of beef cattle, and 0% of transitional swine and small ruminants). Of all positive results, the mean Ct value was 34.28 (SD 3.79), the median Ct value was 35.5, and the range of Ct values was 25.5–39. The one beef cow that tested positive on *lipL32* PCR was from a farm with both dairy (n = 33) and beef (*n* = 2) cattle.

**Figure 4:**
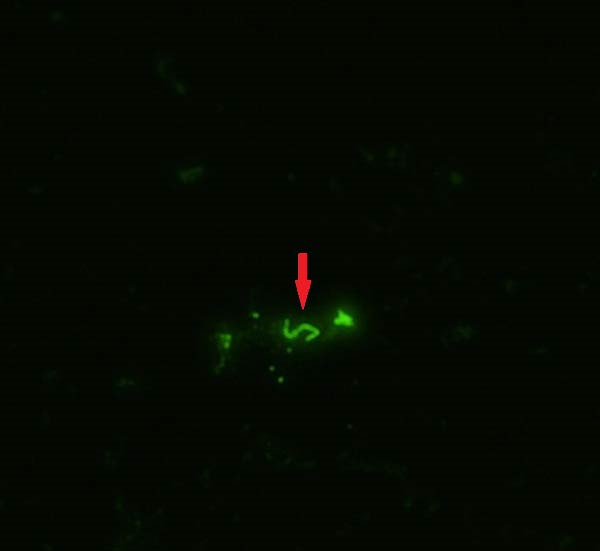
Representative microscopic image (original magnification at 400x, with digital enlargement of 300% during image processing) of a urine sample from dairy cow DCP‐009 sampled in Puerto Rico that was positive for *Leptospira* spp. by FAT. A spirochete *Leptospira* bacterium can be visualized fluorescing in the image, indicated by the red arrow.

Out of the 94 dairy cattle tested, 8.51% (*n* = 8) tested positive for *Leptospira* spp. by FAT (Figure 4), including one individual that tested positive on three sampling days. All beef cattle, transitional swine, and small ruminant samples were negative for *Leptospira* spp. on FAT.

Of all 102 domestic animals with a urine sample tested on both FAT and *lipL32* PCR, 18 (17.65%) were positive on at least one test, but only 5.88% of these animals (*n* = 6, all dairy cattle) tested positive for *Leptospira* spp. on both FAT and *lipL32* PCR.

Feral swine samples tested on *lipL32* PCR include urine samples from eight feral swine and kidney samples from 176 feral swine. Additionally, kidney samples from the two fetuses described earlier, as well as a placental tissue sample from one of the dams, were tested. All samples were negative (Table 1).

**Figure 5:**
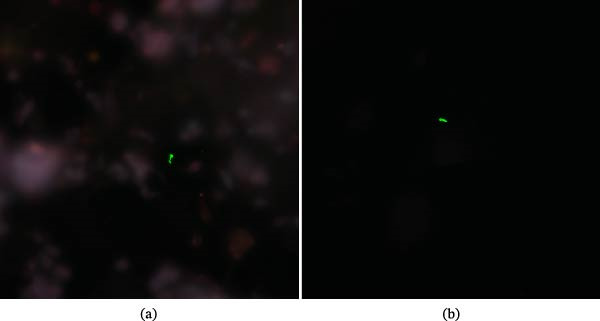
(a) Representative microscopic image (original magnification, 400x) of a urine sample from feral swine CMP‐394 captured in Puerto Rico that was positive for *Leptospira* spp. by FAT. (b) Representative microscopic image (original magnification, 400x) of a urine sample from feral swine CMP‐539 captured in Puerto Rico that was positive for *Leptospira* spp. by FAT. A spirochete *Leptospira* bacterium can be visualized fluorescing in each image.

FAT was performed on a total of 117 urine samples collected from feral swine (Table 1). The urine samples from two animals (1.71%) were positive for *Leptospira* spp. on FAT (Figure 5).

Additional results for urine samples tested on both *lipL32* PCR and FAT are included in Table 4.

**Table 4:** MAT, PCR, and FAT paired leptospirosis results for samples collected from domestic animals and feral swine in Puerto Rico from August 2019 to September 2021.

Type of assay	Animal group	Positive on MAT	Negative on MAT	Positive on urine FAT	Negative on urine FAT
Positive on urine *lipL32* PCR	All domestic animal groups	7/30 (23.33%)	3/30 (10%)	8/113 (7.08%)	15/113 (13.27%)
	Transitional swine	0/0 (0%)	0/0 (0%)	0/1 (0%)	0/1 (0%)
	Dairy cattle	7/25 (28%)	3/25 (12%)	8/105 (7.62%)	14/105 (13.33%)
	Beef cattle	0/4 (0%)	0/4 (0%)	0/6 (0%)	1/6 (16.67%)
	Small ruminants	0/1 (0%)	0/1 (0%)	0/1 (0%)	0/1 (0%)
	Feral swine	0/8 (0%)	0/8 (0%)	0/7 (0%)	0/7 (0%)

Negative on urine *lipL32* PCR	All domestic animal groups	14/30 (46.67%)	6/30 (20%)	2/113 (1.77%)	88/113 (77.88%)
	Transitional swine	0/0 (0%)	0/0 (0%)	0/1 (0%)	1/1 (100%)
	Dairy cattle	10/25 (40.00%)	5/25 (20%)	2/105 (1.90%)	81/105 (77.14%)
	Beef cattle	4/4 (100%)	0/4 (0%)	0/6 (0%)	5/6 (83.33%)
	Small ruminants	0/1 (0%)	1/1 (100%)	0/1 (0%)	1/1 (100%)
	Feral swine	6/8 (75%)	2/8 (25%)	2/7 (28.57%)	5/7 (71.43%)

Positive on urine FAT	All domestic animal groups	1/28 (3.57%)	2/28 (7.14%)	—	—
	Transitional swine	0/0%	0/0 (0%)	—	—
	Dairy cattle	1/23 (4.35%)	2/23 (8.70%)	—	—
	Beef cattle	0/4 (0%)	0/4 (0%)	—	—
	Small ruminants	0/1 (0%)	0/1 (0%)	—	—
	Feral swine	2/110 (1.82%)	0/110 (0%)	—	—

Negative on urine FAT	All domestic animal groups	18/28 (64.29%)	7/28 (25%)	—	—
	Transitional swine	0/0 (0%)	0/0 (0%)	—	—
	Dairy cattle	14/23 (60.87%)	6/23 (26.09%)	—	—
	Beef cattle	4/4 (100%)	0/4 (0%)	—	—
	Small ruminants	0/1 (0%)	1/1 (100%)	—	—
	Feral swine	56/110 (50.91%)	52/110 (47.27%)	—	—

*Note*: Each percentage value represents the percentage of paired samples (for serum and urine sample pairs), or percentage of samples (for urine samples tested on both *lipL32* PCR and FAT) from the given animal group with the specified combination of results. Some animals had multiple sets of paired samples. Locations in the table with dashes do not contain data because they indicate only one diagnostic assay.

### 4.4. *secY* PCR

Of all domestic animals sampled, 4.75% (*n* = 14) of these animals, all of which were dairy cattle with urine samples positive by *lipL32* PCR, had urine samples that were tested by *secY* PCR for *Leptospira* spp. (Table 1). Of these 14 animals, 78.57% (*n* = 11) had positive results on *secY* PCR. From some of these 14 dairy cattle, multiple urine samples were collected, so that 16 individual urine samples were tested on *secY* PCR, 75% (*n* = 12) of which were positive. Amplicon sequencing of *secY* identified nine of these samples from eight dairy cattle as *L. borgpetersenii*, with the remaining three unidentified (Supporting Information 4: File S3).

### 4.5. Urine Culture

Of all 94 dairy cattle with urine samples tested on *lipL32* PCR, 15.96% (*n* = 15) were positive for *Leptospira* spp. (Table 1). As previously described [60], culture was attempted on urine from five dairy cattle with urine samples that were positive on *lipL32* PCR from previous collection days. These five dairy cattle were on three different farms in the municipalities of Gurabo, Quebradillas, and Yabucoa. Out of all cattle for which urine culture was attempted, *Leptospira* spp. were isolated from 60% (*n* = 3). Two of the dairy cows (DCP‐009 and DCP‐017) from which *Leptospira* spp. were isolated were from one farm in Yabucoa, and the third cow (DCP‐041) was from a farm in Quebradillas. Results from serotyping and molecular typing identified the strains isolated from DCP‐009 and DCP‐041 as *L. borgpetersenii* serogroup Sejroe serovar Hardjo. Additionally, the strain isolated from DCP‐017 was identified as *L. santarosai* serogroup Pyrogenes. Serovar determination for the strain isolated from DCP‐017 was not possible due to the inability of serotyping with monoclonal antibodies to differentiate whether it belonged to serovar Alexi, Guaratuba, or Princestown, although it is likely the strain is most closely related to serovar Alexi [60]. Urine culture results were previously described [60].

After *L. borgpetersenii* serogroup Sejroe serovar Hardjo and *L. santarosai* serogroup Pyrogenes were isolated from the dairy cattle, a subsequent study [76] detected co‐infections with multiple *Leptospira* spp. in DCP‐009 and DCP‐017 using a newly‐developed independent DNA capture and enrichment method. Urine from DCP‐009 and DCP‐017 contained both *L. borgpetersenii* and *L. santarosai*.

Urine was cultured from 96 feral swine. Samples from all 96 feral swine exhibited no growth of *Leptospira* isolates (Table 1). Culture was attempted on urine samples from an additional 18 feral swine, but due to sample contamination, culture could not be completed on those samples.

### 4.6. Serial Sampling

**Figure 6:**
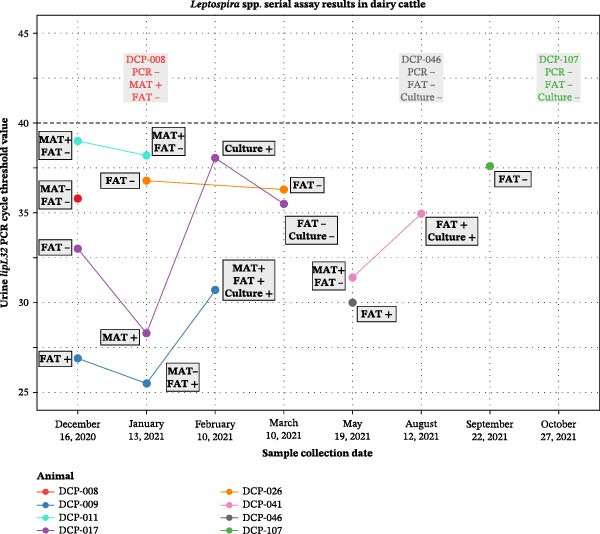
Urine *lipL32* PCR, FAT, culture, and MAT results for *Leptospira* spp. and serogroups for eight dairy cattle from which multiple serum and/or urine samples were collected on multiple days in Puerto Rico from December 2020 to October 2021. PCR Ct values <40 were considered positive. For sample collection days on which urine was negative for *Leptospira* spp. on *lipL32* PCR for an individual animal, any additional MAT, FAT, and/or culture results are described in the text above the Ct cutoff line.

Serial samples were collected from eight dairy cattle on multiple sample collection days. Serial sampling results are demonstrated in Figure 6.

### 4.7. Paired Samples

Thirty pairs of serum and urine samples were collected from a total of 27 domestic animals (22 dairy cattle, four beef cattle, and one ewe). Of these 30 serum and urine sample pairs, 23.33% (*n* = 7) tested positive both on MAT and *lipL32* PCR, respectively. For some dairy cattle, multiple pairs of samples were taken across several days. Further results for paired samples are included in Table 4.

Paired serum and kidney samples were collected and tested from a total of 168 feral swine. Of these animals, 52.98% (*n* = 89) had paired samples with negative serum on MAT and negative kidney on *lipL32* PCR, and 47.02% (*n* = 79) had paired samples with positive serum on MAT and negative kidney on *lipL32* PCR.

A total of 110 pairs of serum and urine samples from feral swine were tested on MAT and FAT, respectively, of which 1.82% (*n* = 2) were positive on both tests. A total of eight pairs of serum and urine samples from feral swine were tested on MAT and *lipL32* PCR, respectively, of which none were positive on both tests. Further results for paired samples are described in Table 4.

### 4.8. Feral Swine and Transitional Swine Combined Data

In total, 543 swine were sampled and tested, including 168 transitional swine and 375 feral swine. Serum samples from 533 swine were tested by MAT, and 60.23% (*n* = 321) were positive for *Leptospira* antibodies.

Among all swine, the *Leptospira* serogroups that were most commonly among the top three highest reacting serogroups in individual animals were serogroup Australis, with antibodies detected in 39.96% (*n* = 213) of swine tested, serogroup Icterohaemorrhagiae, with antibodies detected in 30.77% (*n* = 164) of swine tested, and serogroup Djasiman, with antibodies detected in 17.64% (*n* = 94) of swine tested.

## 5. Discussion

Leptospirosis is an ongoing One Health concern in Puerto Rico and requires continuing research to enhance the understanding of local disease dynamics. To our knowledge, this investigation tested the largest number of domestic livestock and feral swine for *Leptospira* spp. and serogroups in Puerto Rico of any study to date. The results demonstrate that exposure to *Leptospira* spp. and serogroups is common in transitional swine, feral swine, and cattle in specific regions of Puerto Rico.

### 5.1. Importance of Multiple Laboratory Assays

In the diagnosis of leptospirosis, it is essential to perform multiple assays, especially a combination of serologic and molecular assays, in order to obtain the most complete information on *Leptospira* spp. exposure and infection [20, 77]. Depending on the point in the disease process of an infected animal, the *Leptospira* bacterium may be present in some samples and not others [20], thus emphasizing the importance of collecting multiple sample types from an individual. Furthermore, intermittent shedding of the *Leptospira* bacterium has been frequently reported, thus highlighting the importance of collecting samples from a given animal at multiple time points of infection [74].

Of all domestic animals and feral swine with serum and urine samples collected on the same day, many had different results on MAT versus *lipL32* PCR. For the majority of these animals with paired serum and urine samples, positive results on MAT were not complemented by positive results for urine on *lipL32* PCR. This emphasizes that positive MAT results must be interpreted with care, as antibodies detected against a specific *Leptospira* serogroup alone do not indicate active shedding. The timing of sample collection in relation to exposure, as well as the variability of host immune response across sampled individuals, may affect results [20]. Reservoir hosts with chronic infection and detectable antibody titers may demonstrate intermittent shedding of leptospires [74]. Furthermore, negative results on MAT in this study were not always complemented by negative results for urine on *lipL32* PCR, which demonstrates that an animal can be shedding leptospiral DNA with no detectable antibody titers against *Leptospira* serogroups. Evidence of leptospiruria without detectable antibodies has been reported previously, particularly in cattle. Cattle have been shown to excrete *Leptospira* bacteria in urine [78] for months without detectable titers to *Leptospira* serogroups [74, 79]. Multiple explanations exist for negative MAT results in some cases, including very recent exposure, nonspecific agglutination, or delayed or absent seroconversion due to an individual’s unique response to the pathogen [20]. Another possible explanation for these results is that an animal may have antibodies against a specific representative serovar that is not included in the MAT panel being used. The finding of negative serology results and positive *lipL32* PCR results for a specific animal is important for disease surveillance, as it emphasizes the fact that animals that are indeed positive for *Leptospira* spp. and serogroups can test negative for exposure in routine disease surveillance if MAT or another serology assay is the only test that is performed for that individual.

Not only is running multiple diagnostic assays on samples recommended, but it is also essential to test for antibodies to multiple *Leptospira* serovars within a serogroup on an MAT panel. One serovar may be more prevalent within a group of animals than others in the same serogroup [80]. For serogroup Australis, serovars Australis and Bratislava were both included in the MAT panel for all animals tested. Of all detections of antibodies to serogroup Australis on MAT in swine, there were significantly more detections of antibodies to serovar Bratislava than to serovar Australis (Table 3). Additionally, of all detections of antibodies to serogroup Sejroe in dairy cattle, there were significantly more detections of antibodies to serovars Sejroe and Hardjo than to serovar Recreo (Table 3). This emphasizes the importance of testing for multiple serovars within a serogroup in an MAT panel in order to increase the sensitivity of the MAT and to identify a representative reacting serovar for a serogroup in a specific animal population.

Culture is the definitive diagnostic test for identifying infection with transmittable *Leptospira* spp., and isolates can be serotyped and genotyped [20, 78, 81]. Although the time‐intensive culture process necessitates other tests be performed in cases where rapid diagnosis is required, culture isolates can provide the most complete information on the species and serovars commonly infecting animals in a specific region. In this investigation, one dairy cow tested positive on MAT for antibodies against serogroups Sejroe and Australis, and on a consecutive sample day, tested culture positive for *L. santarosai* serogroup Pyrogenes. In this case, serology results did not align with the infecting serogroup as detected by culture. This could in part be explained by cross‐reactivity of MAT antigens to multiple serogroups and serovars, which has been documented previously [20]. Additionally, since the serum for MAT was collected 29 days before the urine culture sample was collected, it is possible that the animal was exposed to serogroup Pyrogenes after the serum sample collection day, or only days before so that sufficient time had not passed to allow for development of detectable antibodies. In other cases in which culture results do not match serology results, there is a possibility that the true infecting serogroup was not included on the MAT antigen panel used to test the serum of a given individual, and was therefore not detected. For these reasons, MAT cannot be used to determine infecting species and serovars, and culture as well as additional molecular diagnostics are needed to do so [20].

The combination of culture results with FAT results in several dairy cattle further highlights the importance of using multiple diagnostic assays when possible. Two dairy cattle (DCP‐009 and DCP‐041) had a given urine sample that tested positive for *Leptospira* spp. on both FAT and culture. In this case, the positive culture results suggest that not only was the bacterium present in the urine samples, but that an active infection with *Leptospira* spp. was ongoing in the individual cattle at the time of sample collection.

Many domestic animals and feral swine in this investigation had different results on *lipL32* PCR and FAT for a given urine sample. This difference was most often (*n* = 15) recorded as a positive result on *lipL32* PCR and a negative result on FAT. Furthermore, three dairy cattle had urine samples that were consistently positive on *lipL32* PCR and negative on FAT across multiple sample days. Discordant results may be explained by the fact that the FAT conjugate used was made from antisera with representatives of serovars Canicola, Grippotyphosa, Hardjo, Icterohaemorrhagiae, and Pomona. From one bovine urine culture, *L. santarosai* serogroup Pyrogenes was isolated, thus highlighting the possibility that all serovars infecting the sampled animals were not represented in the antisera of the FAT conjugate used. The incongruity of *lipL32* PCR and FAT results emphasizes the importance of testing urine samples for *Leptospira* spp. on both PCR and FAT when possible, as they are complimentary tests.

Multiple samples were collected on consecutive sample days from a total of eight dairy cattle. As summarized in Figure 6, several cattle had urine samples collected over time for which the Ct values on *lipL32* PCR varied significantly over time, in one case (DCP‐017) increasing close to the Ct cutoff for detection, and then decreasing again on the next sample day. This emphasizes the well‐documented variable shedding of *Leptospira* spp., particularly in cattle [20, 74]. Leptospires can be shed into the environment via urine for months to years in carrier animals [5, 9, 74, 78, 79], and thus serial sampling provides a more complete understanding of *Leptospira* spp. infection in an animal.

### 5.2. Swine

The detections on MAT of antibodies against serogroups Australis and Djasiman as two of the top three highest reacting serogroups in transitional and feral swine are consistent with previous studies. Domestic swine worldwide [5], as well as feral swine within the United States [27], have been identified as reservoir hosts of serovar Bratislava, a member of the Australis serogroup. Furthermore, antibodies against serogroup Djasiman have been detected in domestic swine in Latin America [82–84] and feral swine in Hawaii [85]. It is important to note that serogroup Djasiman has been identified in environmental water in Iowa and has caused seroconversion without infection in hamsters previously [2]. Thus, further investigation is warranted on differentiating environmental exposure from true infection in swine.

Antibodies against serogroup Cynopteri were among the highest reacting in transitional swine, but not in feral swine. Microagglutinating antibodies against serogroup Cynopteri have seldom been identified in pigs, but have been commonly detected in other species, such as small ruminants, dogs, cats, and humans [86–90], primarily in regions with subtropical and tropical climates. Due to the multiple studies detecting microagglutinating antibodies against serogroup Cynopteri in cats, as well as the large populations of free‐roaming cats in rural areas of Puerto Rico, particularly around farms, it is possible that cats play a role in leptospirosis epidemiology in transitional swine in backyard farms. Therefore, further investigation is warranted to determine how various animal reservoirs may play a role in disease transmission.

In feral swine, all urine and kidney samples tested negative on *lipL32* PCR. According to previous studies, *Leptospira* spp. can typically begin to be detected in the urine on average 7 days after *Leptospira* spp. are first detectable in blood [20]. Given the high seroprevalence of agglutinating antibodies to *Leptospira* serogroups in this feral swine study population, it is likely that feral swine with positive MAT results and negative *lipL32* PCR results were exposed to *Leptospira* spp. long before sampling. *Leptospira* antibodies can remain in an animal’s serum for months to years after initial exposure [20]; therefore, it is possible for animals to have circulating antibodies after fully clearing an infection with *Leptospira* spp. This is further supported by the fact that the mean and median MAT titers of *Leptospira* antibodies were relatively low in feral swine, which may indicate a historical exposure versus a recent exposure [27, 91]. It is also possible that, in those cases of feral swine with positive MAT results and negative *lipL32* PCR results, the animal was infected by *Leptospira* spp. at the time of sample collection but was either not shedding *Leptospira* spp. at the time of sample collection, or was doing so at undetectable levels. However, there were two feral swine with serum samples that tested positive on MAT and urine samples that tested positive on FAT, which again highlights the importance of testing urine samples on PCR and FAT when possible.

From transitional sow TSP‐0350, the kidney was positive for *Leptospira* spp. on *lipL32* PCR, but the whole blood was negative on *lipL32* PCR. It has been noted that leptospiremia is often brief and occurs only at the beginning of infection, often lasting anywhere from 2–20 days post‐exposure [20], although leptospiremia most often lasts less than a week [92]. Therefore, it is possible that *Leptospira* spp. had become established in the kidneys and were no longer present in the blood at the time of sample collection.

### 5.3. Cattle

The finding of antibodies against serogroup Sejroe as the highest reacting antibodies in cattle suggests likely exposure of the animals to this serogroup. Furthermore, in this investigation, *L. borgpetersenii* serogroup Sejroe serovar Hardjo was isolated from the urine samples of two dairy cattle, one of which was seropositive during the study period for antibodies against serogroup Sejroe serovar Hardjo. This is consistent with previous studies demonstrating that cattle are reservoir hosts of serogroup Sejroe serovar Hardjo [5], often excreting leptospires in the urine [78, 93]. Cattle have been identified as chronic shedders of both *L. borgpetersenii* serovar Hardjo (type Hardjobovis) and *L. interrogans* serovar Hardjo (type Hardjoprajitno) [94, 95], which are indistinguishable on serology but are distinct on molecular typing. Cattle are known globally to be reservoirs of *L. borgpetersenii* [74, 96–98], and can transmit the bacterium to humans and animals [5].

Additional culture results of *L. santarosai* serogroup Pyrogenes in another dairy cow are noteworthy, as *L. santarosai* often causes infection in humans, as well as domestic and wild animals across Latin America [99–104]. *L. santarosai* has been isolated from human patients in Puerto Rico [35], thus emphasizing the potential for zoonotic transmission of *L. santarosai*.

The finding that antibodies against serogroups Hebdomadis and Mini were among the highest reacting antibodies is consistent with previous studies reporting antibodies to these serogroups in cattle worldwide [105–111].

Multiple dairy cattle with serial urine samples demonstrated long‐lasting bacterial shedding, with shedding time frames lasting 56, 84, and 85 days, depending on the individual. Because urine samples were not collected and tested on every day during these time frames, it is possible that one or more of these animals were intermittently shedding *Leptospira* spp. between sample collection days. However, the fact that the animals were still shedding leptospires in the urine 56, 84, and 85 days after their urine was initially positive on *lipL32* PCR demonstrates that cattle are capable of shedding leptospires into the environment for long periods of time as reservoir hosts, serving as possible point sources for transmission to other animals within and outside their species and to humans. These findings are consistent with previous reports of prolonged leptospiruria in cattle, lasting from 79 to 259 days [74, 79].

### 5.4. Outreach

In order to address the significant public health risk that *Leptospira* spp. pose to farms where animals in this investigation have been identified to be infected with *Leptospira* spp., outreach was conducted. USDA APHIS VS met with producers to discuss the results of *Leptospira* spp. testing in their animals and how the findings are relevant to the health of humans and animals on the farm. An informational document was created in both Spanish and English (Supporting Information 1: Figure S1) and distributed to inform producers of important leptospirosis details, including how to prevent transmission to themselves and to their employees.

### 5.5. Limitations

Several limitations should be considered when interpreting the findings of this investigation. Temporal trend analysis of infection and seropositivity was not conducted. It is well‐documented that seasonal variations in weather, as well as sudden variations in weather due to extreme events, such as hurricanes, can impact *Leptospira* spp. transmission dynamics. However, due to constraints and investigation scope, sample sizes across different weather events were not sufficient to support statistical analysis of temporal trends. Therefore, it is recommended that future leptospirosis studies analyze the temporal trends of transmission dynamics. Furthermore, because of the opportunistic nature of this study, samples were collected in conjunction with USDA APHIS activities, and were not collected evenly across all geographic regions in Puerto Rico. Because samples were not collected through a random or systematic sampling design, this may have led to over or under‐representation of disease‐prone areas. This limits the applicability of investigation findings to interpretations of island‐wide *Leptospira* distribution. It is recommended that future studies involve sample collection across wider geographies of Puerto Rico.

### 5.6. Prevention and Preparedness Measures

In order to best prevent and prepare for future outbreaks of leptospirosis in humans and animals in Puerto Rico, it is essential to conduct further investigations of *Leptospira* spp. in a wider range of host species across a larger geographic area. The complex interaction between the environment, reservoir hosts, and susceptible hosts in *Leptospira* etiology, as well as the wide variety of pathogenic *Leptospira* spp. and serogroups, make having current, comprehensive information vital to developing disease prevention and preparedness plans. Additionally, further investigations assessing the role of reservoir species and the environment in disease transmission are needed. In future investigations, not only serology, but also molecular assays, must be performed to identify exposure to *Leptospira* spp., as well as to determine the species and serogroups involved in current infection.

Many commercially available vaccines for animals against *Leptospira* spp. are polyvalent inactivated vaccines and contain bacterins specific for the serovars found to be prevalent at the time of vaccine development in the targeted species and region of the world. This investigation will inform more effective, targeted vaccine development and vaccination implementation based on prevalent serovars. Additionally, a more thorough understanding of circulating *Leptospira* spp. and serogroups will aid in the development of MAT antigen panels that are specific to those serovars circulating in Puerto Rico.

## 6. Conclusions

The presence of reservoir hosts, in addition to flooding events often associated with hurricanes, have likely contributed to the prevalence of *Leptospira* spp. infection and exposure in Puerto Rico as detected in this investigation. The isolation of *L. borgpetersenii* serogroup Sejroe serovar Hardjo and *L. santarosai* serogroup Pyrogenes from dairy cattle in Puerto Rico, as well as the identification of frequently detected antibodies against specific *Leptospira* serogroups, provides insight on the circulating species, serogroups, and serovars in specific regions of Puerto Rico.

The results of this investigation will inform future disease preparedness, response, and mitigation efforts in Puerto Rico. This investigation has demonstrated that a large number of domestic animals and feral swine have been exposed to leptospirosis throughout various regions of Puerto Rico. Livestock producers are often in close proximity with the animals, and therefore, risk of zoonotic spread of leptospirosis from livestock to humans is high. To prevent and prepare for outbreaks in humans and animals, public outreach efforts must be continued to further inform livestock producers of the zoonotic potential of *Leptospira* spp., particularly in the regions where a large number of exposed animals were detected. This outreach should involve providing biosecurity recommendations for farmers to further empower them to not only prevent outbreaks, but to quickly contain an outbreak when it does occur, thus mitigating the number of humans and animals impacted.

## Funding

This research was supported in part by U.S. Department of Agriculture Animal and Plant Health Inspection Service and Centers for Disease Control and Prevention supplemental congressional funding in response to 2017 Hurricanes Irma and Maria. The U.S. Department of Agriculture Animal and Plant Health Inspection Service, Wildlife Services National Feral Swine Damage Management Program funded the operational activities associated with feral swine removal. A portion of the laboratory work was funded by an appointment to the Animal and Plant Health Inspection Service Research Participation Program administered by the Oak Ridge Institute for Science and Education (ORISE) through an interagency agreement between the U.S. Department of Energy (DOE) and the U.S. Department of Agriculture. ORISE is managed by Oak Ridge Associated Universities under DOE Contract Number DESC0014664. This research did not receive any specific grant from funding agencies in the public, commercial, or not‐for‐profit sectors.

## Disclosure

The findings and conclusions in this publication are those of the authors and do not necessarily represent the official position of CDC, USDA, or U.S. Government.

## Conflicts of Interest

The authors declare no conflicts of interest.

## Supporting Information

Additional supporting information can be found online in the Supporting Information section.

## Supporting information


**Supporting Information 1** Table S1. ZSAL and NCAH microscopic agglutination test antigen panels for *Leptospira* serogroup testing of domestic animal and feral swine samples collected in Puerto Rico from August 2019 to September 2021. Figure S1. Leptospirosis outreach document provided to owners of animals involved in the investigation of leptospirosis in domestic livestock and feral swine in Puerto Rico from August 2019 to September 2021 (English and Spanish versions provided).

**Supporting Information 2** File S1. Leptospirosis testing results (MAT, PCR, FAT, and culture) for domestic animals sampled in Puerto Rico from September 2019 to September 2021.

**Supporting Information 3** File S2. Leptospirosis testing results (MAT, PCR, FAT, and culture) for feral swine sampled in Puerto Rico from August 2019 to June 2021.

**Supporting Information 4** File S3. *secY* PCR sequencing results for dairy cattle urine samples collected in Puerto Rico during the investigation of leptospirosis in domestic livestock and feral swine from August 2019 to September 2021.

## Data Availability

The data that support the findings of this study are available in the Supporting Information section of this article.

## References

[1] Sohm C. , Steiner J. , and Jöbstl J. , et al.A Systematic Review on Leptospirosis in Cattle: A European Perspective, One Health. (2023) 17, 10.1016/j.onehlt.2023.100608, 100608. 37577054 PMC10416059

[2] Hamond C. , LeCount K. , and Anderson T. , et al.Isolation and Characterization of Saprophytic and Pathogenic Strains of Leptospira From Water Sources in the Midwestern United States, Frontiers in Water. (2024) 6, 10.3389/frwa.2024.1278088, 1278088.

[3] Hamond C. , Tibbs-Cortes B. , and Fernandes L. G. , et al.Leptospira Gorisiae Sp, Nov: Five New Species Isolated From Water Sources in the Midwestern United States, International Journal of Systematic and Evolutionary Microbiology. (2025) 75, no. 1, 006595. 39773342 10.1099/ijsem.0.006595 PMC11706286

[4] Picardeau M. , Virulence of the Zoonotic Agent of Leptospirosis: Still Terra Incognita?, Nature Reviews Microbiology. (2017) 15, no. 5, 297–307, 10.1038/nrmicro.2017.5, 2-s2.0-85014563167. 28260786

[5] Anna Rovid Spickler KLL. , Leptospirosis, 2013.

[6] Karpagam K. B. and Ganesh B. , Leptospirosis: A Neglected Tropical Zoonotic Infection of Public Health Importance—An Updated Review, European Journal of Clinical Microbiology & Infectious Diseases. (2020) 39, no. 5, 835–846, 10.1007/s10096-019-03797-4. 31898795

[7] Costa F. , Hagan J. E. , and Calcagno J. , et al.Global Morbidity and Mortality of Leptospirosis: A Systematic Review, PLoS Neglected Tropical Diseases. (2015) 9, no. 9, 10.1371/journal.pntd.0003898, 2-s2.0-84943161123. PMC4574773 26379143

[8] Sanders E. J. , Rigau-Pérez J. G. , and Smits H. L. , et al.Increase of Leptospirosis in Dengue-Negative Patients After a Hurricane in Puerto Rico in 1996, The American Journal of Tropical Medicine and Hygiene. (1999) 61, no. 3, 399–404, 10.4269/ajtmh.1999.61.399, 2-s2.0-0345411404. 10497979

[9] Bharti A. R. , Nally J. E. , and Ricaldi J. N. , et al.Leptospirosis: A Zoonotic Disease of Global Importance, The Lancet Infectious Diseases. (2003) 3, no. 12, 757–771, 10.1016/S1473-3099(03)00830-2, 2-s2.0-0344983453. 14652202

[10] Cousins D. , Ellis T. , Parkinson J. , and McGlashan C. , Evidence for Sheep as a Maintenance Host for *Leptospira interrogans* Serovar Hardjo, 1989. 10.1136/vr.124.5.123 2922905

[11] Gerritsen M. J. , Koopmans M. J. , Peterse D. , and Olyhoek T. , Sheep as Maintenance Host for *Leptospira interrogans* Serovar Hardjo Subtype Hardjobovis, American Journal of Veterinary Research. (1994) 55, no. 9, 1232–1237, 10.2460/ajvr.1994.55.09.1232. 7802389

[12] Andreani E. , Tolari F. , and Farina R. , Experimental Infection in Sheep With *Leptospira interrogans* Serotype Hardjo, British Veterinary Journal. (1983) 139, no. 2, 165–170, 10.1016/S0007-1935(17)30540-7, 2-s2.0-0020724124. 6839124

[13] Farina R. , Cerri D. , and Renzoni G. , et al. *Leptospira interrogans* in the Genital Tract of Sheep. Research on Ewes and Rams Experimentally Infected With Serovar Hardjo (hardjobovis), The New Microbiologica. (1996) 19, no. 3, 235–242. 8841039

[14] Hamond C. , Adam E. N. , and Stone N. E. , et al.Identification of Equine Mares as Reservoir Hosts for Pathogenic Species of Leptospira, Frontiers in Veterinary Science. (2024) 11, 10.3389/fvets.2024.1346713, 1346713. 38784659 PMC11112012

[15] Martins G. and Lilenbaum W. , Leptospirosis in Sheep and Goats under Tropical Conditions, Tropical Animal Health and Production. (2014) 46, no. 1, 11–17, 10.1007/s11250-013-0480-6, 2-s2.0-84898756414. 24085419

[16] Lilenbaum W. , Varges R. , and Ristow P. , et al.Identification of Leptospira Spp. Carriers Among Seroreactive Goats and Sheep by Polymerase Chain Reaction, Research in Veterinary Science. (2009) 87, no. 1, 16–19, 10.1016/j.rvsc.2008.12.014, 2-s2.0-67349228539. 19232418

[17] Tripathy D. , Hanson L. , Mansfield M. , and Thilsted J. , Experimental Infection of Lactating Goats With *Leptospira interrogans* Serovars Pomona and Hardjo, American Journal of Veterinary Research. (1985) 46, no. 12, 2512–2514, 10.2460/ajvr.1985.46.12.2512. 4083585

[18] Tilahun Z. , Reta D. , and Simenew K. , Global Epidemiological Overview of Leptospirosis, International Journal of Microbiological Research. (2013) 4, no. 1, 5–9.

[19] Yadeta W. , Bashahun G. , and Abdela N. , Leptospirosis in Animal and its Public Health Implications: A Review, World Applied Sciences Journal. (2016) 34, no. 6, 845–853.

[20] Sykes J. E. , Reagan K. L. , Nally J. E. , Galloway R. L. , and Haake D. A. , Role of Diagnostics in Epidemiology, Management, Surveillance, and Control of Leptospirosis, Pathogens. (2022) 11, no. 4, 10.3390/pathogens11040395, 395. 35456070 PMC9032781

[21] Trueba G. , Zapata S. , Madrid K. , Cullen P. , and Haake D. , Cell Aggregation: A Mechanism of Pathogenic Leptospira to Survive in Fresh Water, International Microbiology. (2004) 7, no. 1, 35–40. 15179605

[22] Zuerner R. L. , Cameron C. E. , and Raverty S. , et al.Geographical Dissemination of *Leptospira interrogans* Serovar Pomona During Seasonal Migration of California Sea Lions, Veterinary Microbiology. (2009) 137, no. 1-2, 105–110, 10.1016/j.vetmic.2008.12.017, 2-s2.0-67349144133. 19186009

[23] Cilia G. , Bertelloni F. , Albini S. , and Fratini F. , Insight Into the Epidemiology of Leptospirosis: A Review of Leptospira Isolations From “Unconventional” Hosts, Animals. (2021) 11, no. 1, 10.3390/ani11010191, 191. 33466962 PMC7830643

[24] Prager K. C. , Greig D. J. , and Alt D. P. , et al.Asymptomatic and Chronic Carriage of *Leptospira interrogans* Serovar Pomona in California Sea Lions (*Zalophus californianus*), Veterinary Microbiology. (2013) 164, no. 1-2, 177–183, 10.1016/j.vetmic.2013.01.032, 2-s2.0-84875819512. 23419822

[25] Benavidez K. M. , Guerra T. , and Torres M. , et al.The Prevalence of Leptospira Among Invasive Small Mammals on Puerto Rican Cattle Farms, PLoS Neglected Tropical Diseases. (2019) 13, no. 5, 10.1371/journal.pntd.0007236, 2-s2.0-85066965077. PMC6544380 31107872

[26] Briskin E. A. , Casanovas-Massana A. , and Ryff K. R. , et al.Seroprevalence, Risk Factors, and Rodent Reservoirs of Leptospirosis in an Urban Community of Puerto Rico, 2015, The Journal of Infectious Diseases. (2019) 220, no. 9, 1489–1497, 10.1093/infdis/jiz339, 2-s2.0-85072717137. 31342075 PMC6761939

[27] Pedersen K. , Pabilonia K. , and Anderson T. , et al.Widespread Detection of Antibodies to Leptospira in Feral Swine in the United States, Epidemiology & Infection. (2015) 143, no. 10, 2131–2136, 10.1017/S0950268814003148, 2-s2.0-84930759245. 25518910 PMC9506984

[28] Barcellos C. and Sabroza P. C. , The Place Behind the Case: Leptospirosis Risks and Associated Environmental Conditions in a Flood-Related Outbreak in Rio de Janeiro, Cadernos de Saúde Pública. (2001) 17, no. suppl, S59–S67, 10.1590/S0102-311X2001000700014. 11426266

[29] Ko A. I. , Reis M. G. , Dourado C. M. R. , Johnson W. D. , and Riley L. W. , Urban Epidemic of Severe Leptospirosis in Brazil, The Lancet. (1999) 354, no. 9181, 820–825, 10.1016/S0140-6736(99)80012-9, 2-s2.0-0033523415. 10485724

[30] Maciel E. A. , de Carvalho A. L. F. , and Nascimento S. F. , et al.Household Transmission of Leptospira Infection in Urban Slum Communities, PLoS Neglected Tropical Diseases. (2008) 2, no. 1, 10.1371/journal.pntd.0000154, 2-s2.0-48949117995. PMC2270796 18357340

[31] Bacallao J. , Schneider M. C. , and Najera P. , et al.Socioeconomic Factors and Vulnerability to Outbreaks of Leptospirosis in Nicaragua, International Journal of Environmental Research & Public Health. (2014) 11, no. 8, 8301–8318, 10.3390/ijerph110808301, 2-s2.0-84926078329. 25153463 PMC4143863

[32] Hart R. , Gallagher J. , and Waitkins S. , An Outbreak of Leptospirosis in Cattle and Man, BMJ. (1984) 288, no. 6435, 1983–1984, 10.1136/bmj.288.6435.1983. 6428630 PMC1442176

[33] Robertson M. , Clarke I. , Coghlan J. , and Gill O. , Leptospirosis in Trout Farmers, The Lancet. (1981) 318, no. 8247, 626–627, 10.1016/S0140-6736(81)92757-4, 2-s2.0-0019521060. 6116098

[34] Everard C. O. , Edwards C. N. , Everard J. D. , and Carrington D. G. , A Twelve-Year Study of Leptospirosis on Barbados, European Journal of Epidemiology. (1995) 11, no. 3, 311–320, 10.1007/BF01719436, 2-s2.0-0028981342. 7493664

[35] Alexander A. , Leptospirosis in Puerto Rico, 1963. 14170551

[36] Ramos L. J. S. , Lázaro P. M. , Barbosa J. S. , and Cardona C. P. , Análisis Espacial y Epidemiológico Sobre Leptospirosis Humana en Puerto Rico, 1996 a 2014, GeoFocus International Review of Geographical Information Science and Technology. (2018) 21, 227–251.

[37] Smythe L. , Dohnt M. , and Symonds M. , et al.Review of Leptospirosis Notifications in Queensland and Australia: January 1998-June 1999, Communicable Diseases Intelligence. (2000) 24, no. 6, 153–157, 10.33321/cdi.2000.24.23. 10943028

[38] Desai K. , Patel F. , Patel P. , Nayak S. , Patel N. , and Bansal R. , A Case–Control Study of Epidemiological Factors Associated With Leptospirosis in South Gujarat Region, Journal of Postgraduate Medicine. (2016) 62, no. 4, 223–227, 10.4103/0022-3859.188551, 2-s2.0-84994589896. 27763478 PMC5105206

[39] Gill O. N. , Coghlan J. D. , and Calder I. M. , The Risk of Leptospirosis in United Kingdom Fish Farm Workers. Results From a 1981 Serological Survey, Journal of Hygiene. (1985) 94, no. 1, 81–86, 10.1017/S0022172400061155, 2-s2.0-0021928119. 3973382 PMC2129388

[40] Campagnolo E. R. , Warwick M. C. , and Marx H. L. , et al.Analysis of the 1998 Outbreak of Leptospirosis in Missouri in Humans Exposed to Infected Swine, Journal of the American Veterinary Medical Association. (2000) 216, no. 5, 676–682, 10.2460/javma.2000.216.676, 2-s2.0-0034145464. 10707682

[41] Chan O. Y. , Paul D. R. , and Sng E. H. , Leptospirosis Among Abattoir Workers—A Serological Survey, Singapore Medical Journal. (1987) 28, no. 4, 293–296. 3423792

[42] Terry J. , Trent M. , and Bartlett M. , Cluster of Leptospirosis Among Abattoir Workers, Communicable Diseases Intelligence. (2000) 24, no. 6, 158–160, 10.33321/cdi.2000.24.24. 10943029

[43] Blackmore D. K. , Bell L. , and Schollum L. , Leptospirosis in Meat Inspectors: Preliminary Results of a Serological Survey, The New Zealand Medical Journal. (1979) 90, no. 648, 415–418. 293569

[44] Şükran K. , Tatar B. , Ersan G. , and Topaloğlu S. , A Leptospirosis Case Presenting With Thrombotic Thrombocytopenic Purpura, Balkan Medical Journal. (2013) 2013, no. 4, 436–438. 10.5152/balkanmedj.2013.9078 PMC4115946 25207155

[45] Abd Rahim M. A. , Zaki A. M. , and Atil A. , et al.Effectiveness of Antibiotic Prophylaxis for Leptospirosis Among Adults: A Systematic Review, Malaysian Journal of Applied Sciences. (2018) 3, no. 2, 46–56.

[46] Sehgal S. C. , Sugunan A. P. , and Vijayachari P. , Outbreak of Leptospirosis After the Cyclone in Orissa, National Medical Journal of India. (2002) 15, no. 1, 22–23. 11855587

[47] Liverpool J. , Francis S. , Liverpool C. E. , Dean G. T. , and Mendez D. D. , Leptospirosis: Case Reports of an Outbreak in Guyana, Annals of Tropical Medicine & Parasitology. (2008) 102, no. 3, 239–245, 10.1179/136485908X278784, 2-s2.0-44449143631. 18348778

[48] Amilasan A. S. , Ujiie M. , and Suzuki M. , et al.Outbreak of Leptospirosis After Flood, the Philippines, 2009, Emerging Infectious Diseases. (2012) 18, no. 1, 91–94, 10.3201/eid1801.101892, 2-s2.0-84863012315. 22257492 PMC3310081

[49] Schneider M. C. , Nájera P. , and Aldighieri S. , et al.Leptospirosis Outbreaks in Nicaragua: Identifying Critical Areas and Exploring Drivers for Evidence-Based Planning, International Journal of Environmental Research and Public Health. (2012) 9, no. 11, 3883–3910, 10.3390/ijerph9113883, 2-s2.0-84870846062. 23202822 PMC3524603

[50] Weinberger D. , Baroux N. , Grangeon J.-P. , Ko A. I. , and Goarant C. , El Niño Southern Oscillation and Leptospirosis Outbreaks in New Caledonia, PLoS Neglected Tropical Diseases. (2014) 8, no. 4, 10.1371/journal.pntd.0002798, 2-s2.0-84901266617. PMC3990495 24743322

[51] Schneider M. C. , Najera P. , and Pereira M. M. , et al.Leptospirosis in Rio Grande do Sul, Brazil: An Ecosystem Approach in the Animal-Human Interface, PLoS Neglected Tropical Diseases. (2015) 9, no. 11, 10.1371/journal.pntd.0004095, 2-s2.0-84949502358. PMC4643048 26562157

[52] Kawaguchi L. , Sengkeopraseuth B. , and Tsuyuoka R. , et al.Seroprevalence of Leptospirosis and Risk Factor Analysis in Flood-Prone Rural Areas in Lao PDR, The American Journal of Tropical Medicine and Hygiene. (2008) 78, no. 6, 957–961, 10.4269/ajtmh.2008.78.957. 18541776

[53] Chapman T. , Bachoon D. S. , Martinez G. A. , Burt C. D. , and DeMontigny W. C. , Tracking the Sources of Leptospira and Nutrient Flows in Two Urban Watersheds of Puerto Rico, Environmental Monitoring and Assessment. (2023) 195, no. 11, 10.1007/s10661-023-11948-6, 1318. 37833564

[54] Stone N. E. , Hall C. M. , and Ortiz M. , et al.Diverse Lineages of Pathogenic Leptospira Species are Widespread in the Environment in Puerto Rico, USA, PLoS Neglected Tropical Diseases. (2022) 16, no. 5, 10.1371/journal.pntd.0009959. PMC9154103 35584143

[55] Jones F. K. , Ryff A. G. M. , and Irizarry-Ramos J. , et al. Marzán-Rodriguez , Leptospirosis Outbreak After Hurricane Fiona, Puerto Rico, 2022, *EIS Conference*, 2023. 10.15585/mmwr.mm7335a2 PMC11376507 39236025

[56] Ryff K. R. , Rivera A. , and Rodriguez D. M. , et al.Epidemiologic Trends of Dengue in US Territories, 2010-2020, MMWR. Surveillance Summaries. (2023) 72, no. 4, 1–12, 10.15585/mmwr.ss7204a1. PMC10208330 37192141

[57] Sheffield P. , Rowe M. , Agu D. , Rodríguez L. , and Avilés K. , Health Impact Assessments for Environmental Restoration: The Case of Caño Martín Peña, Annals of Global Health. (2014) 80, no. 4, 296–302, 10.1016/j.aogh.2014.07.001, 2-s2.0-84927668358. 25459331 PMC4268865

[58] ENLACE (Corporación del Proyecto ENLACE del Caño Martín Peña) , Socioeconomic Profile: Caño Martín Peña Special Planning District. Factsheet., 2010.

[59] Fernandes L. G. , Stone N. E. , and Roe C. C. , et al.Leptospira Sanjuanensis Sp. Nov., a Pathogenic Species of the Genus Leptospira Isolated From Soil in Puerto Rico, International Journal of Systematic and Evolutionary Microbiology. (2022) 72, no. 10, 10.1099/ijsem.0.005560, 005560. 36260655

[60] Hamond C. , Dirsmith K. L. , and LeCount K. , et al. *Leptospira borgpetersenii* Serovar Hardjo and *Leptospira santarosai* Serogroup Pyrogenes Isolated From Bovine Dairy Herds in Puerto Rico, Frontiers in Veterinary Science. (2022) 9, 10.3389/fvets.2022.1025282, 1025282. 36467637 PMC9712998

[61] Hornsby R. L. , Alt D. P. , and Nally J. E. , Isolation and Propagation of Leptospires at 37 °C Directly From the Mammalian Host, Scientific Reports. (2020) 10, no. 1, 10.1038/s41598-020-66526-4, 9620. 32541841 PMC7296004

[62] Ellis W. , Montgomery J. , and Cassells J. , Dihydrostreptomycin Treatment of Bovine Carriers of *Leptospira interrogans* Serovar Hardjo, Research in Veterinary Science. (1985) 39, no. 3, 292–295, 10.1016/S0034-5288(18)31716-8. 2417297

[63] Reiland S. , Growth and Skeletal Development of the Pig, Acta Radiologica. Supplementum. (1978) 358, 15–22. 233594

[64] R Development Core Team , R: A Language and Environment for Statistical Computing, R Foundation for Statistical Computing, 2023.

[65] Posit Team , RStudio: Integrated Development Environment for R, 2023, Posit Software.

[66] Wickham H. , Bryan A. M. , and Chang W. , et al.Welcome to the tidyverse, Journal of Open Source Software. (2019) 4, e1686.

[67] Wickham H. , ggplot2: Elegant Graphics for Data Analysis, 2016, Springer-Verlag New York.

[68] Cole J. R. , Sulzer C. R. , and Pursell A. R. , Improved Microtechnique for the Leptospiral Microscopic Agglutination Test, Applied Microbiology. (1973) 25, no. 6, 976–980, 10.1128/am.25.6.976-980.1973. 4736794 PMC380950

[69] Galloway R. L. and Hoffmaster A. R. , Optimization of LipL32 PCR Assay for Increased Sensitivity in Diagnosing Leptospirosis, Diagnostic Microbiology and Infectious Disease. (2015) 82, no. 3, 199–200, 10.1016/j.diagmicrobio.2015.03.024, 2-s2.0-84941878077. 25912810 PMC6452440

[70] Stoddard R. A. , Gee J. E. , Wilkins P. P. , McCaustland K. , and Hoffmaster A. R. , Detection of Pathogenic *Leptospira* Spp. Through TaqMan Polymerase Chain Reaction Targeting the LipL32 Gene, Diagnostic Microbiology and Infectious Disease. (2009) 64, no. 3, 247–255, 10.1016/j.diagmicrobio.2009.03.014, 2-s2.0-67349163028. 19395218

[71] Hamond C. , Browne A. S. , and de Wilde L. H. , et al.Assessing Rodents as Carriers of Pathogenic Leptospira Species in the US Virgin Islands and Their Risk to Animal and Public Health, Scientific Reports. (2022) 12, no. 1, 10.1038/s41598-022-04846-3, 1132. 35064157 PMC8782869

[72] Ahmed N. , Devi S. M. , and De los Á Valverde , et al.Multilocus Sequence Typing Method for Identification and Genotypic Classification of Pathogenic Leptospira Species, Annals of Clinical Microbiology and Antimicrobials. (2006) 5, 1–10, 10.1186/1476-0711-5-28, 2-s2.0-34547645281. 17121682 PMC1664579

[73] Ahmed A. , Engelberts M. F. M. , Boer K. R. , Ahmed N. , Hartskeerl R. A. , and Bereswill S. , Development and Validation of a Real-Time PCR for Detection of Pathogenic Leptospira Species in Clinical Materials, PLoS ONE. (2009) 4, no. 9, 10.1371/journal.pone.0007093, 2-s2.0-70349442462. PMC2740861 19763264

[74] Hamond C. , LeCount K. , and Putz E. J. , et al.Bovine Leptospirosis due to Persistent Renal Carriage of *Leptospira borgpetersenii* Serovar Tarassovi, Frontiers in Veterinary Science. (2022) 9, 10.3389/fvets.2022.848664, 848664. 35464389 PMC9019706

[75] Hartskeerl R. , Smits H. , Korver H. , Goris M. , and Terpstra W. , Manual International Course on Laboratory Methods for the Diagnosis of Leptospirosis, 2006, KIT.

[76] Stone N. E. , McDonough R. F. , and Hamond C. , et al.DNA Capture and Enrichment: A Culture-Independent Approach for Characterizing the Genomic Diversity of Pathogenic Leptospira Species, Microorganisms. (2023) 11, no. 5, 10.3390/microorganisms11051282, 1282. 37317256 PMC10224534

[77] Wagenaar J. , Zuerner R. L. , Alt D. , and Bolin C. A. , Comparison of Polymerase Chain Reaction Assays With Bacteriologic Culture, Immunofluorescence, and Nucleic Acid Hybridization for Detection of *Leptospira borgpetersenii* Serovar Hardjo in Urine of Cattle, American Journal of Veterinary Research. (2000) 61, no. 3, 316–320, 10.2460/ajvr.2000.61.316, 2-s2.0-0034146160. 10714525

[78] Nally J. E. , Hornsby R. L. , and Alt D. P. , et al.Isolation and Characterization of Pathogenic Leptospires Associated With Cattle, Veterinary Microbiology. (2018) 218, 25–30, 10.1016/j.vetmic.2018.03.023, 2-s2.0-85044467933. 29685217

[79] Monti G. , Montes V. , Tortosa P. , Tejeda C. , and Salgado M. , Urine Shedding Patterns of Pathogenic Leptospira Spp. in Dairy Cows, Veterinary Research. (2023) 54, no. 1, 10.1186/s13567-023-01190-w, 64. 37525220 PMC10391894

[80] Arent Z. , Andrews S. , Adamama-Moraitou K. , Gilmore C. , Pardali D. , and Ellis W. , Emergence of Novel Leptospira Serovars: A Need for Adjusting Vaccination Policies for Dogs?, Epidemiology & Infection. (2013) 141, no. 6, 1148–1153, 10.1017/S0950268812002087, 2-s2.0-84870562667. 22998981 PMC9151848

[81] Miller D. A. , Wilson M. A. , and Beran G. W. , Survey to Estimate Prevalence of *Leptospira interrogans* Infection in Mature Cattle in the United States, American Journal of Veterinary Research. (1991) 52, no. 11, 1761–1765, 10.2460/ajvr.1991.52.11.1761. 1785719

[82] Rauber L. , de Cesaro Cavaler A. , and de Araujo G. V. , et al.Soroprevalência de leptospirose suína na região noroeste do Paraná, Arquivos de Ciências Veterinárias e Zoologia da UNIPAR. (2011) 14, no. 1, 33–35.

[83] Cruz-Romero A. , Alvarado-Esquivel C. , and Romero-Salas D. , et al.Seroepidemiology of Leptospira Infection in Backyard Pigs in Durango State, Mexico, European Journal of Microbiology and Immunology. (2018) 8, no. 3, 87–90, 10.1556/1886.2018.00009. 30345088 PMC6186016

[84] Samico-Fernandes E. F. T. , de Albuquerque P. P. F. , and Fernandes M. F. T. S. , et al.Anti-Leptospira Spp. Antibodies in Pigs Slaughtered in the Agreste Region of Pernambuco, Brazil, Acta Scientiae Veterinariae. (2019) 47, no. 1, 10.22456/1679-9216.93772.

[85] Buchholz A. E. , Katz A. R. , Galloway R. , Stoddard R. A. , and Goldstein S. M. , Feral Swine Leptospira Seroprevalence Survey in Hawaii, USA. 2007-2009, Zoonoses and Public Health. (2016) 63, no. 8, 584–587, 10.1111/zph.12266, 2-s2.0-84960330201. 26969849 PMC6374768

[86] Levett P. N. , Whittington C. , and Camus E. , Serological Survey of Leptospirosis in Livestock Animals in the Lesser Antilles, Annals of the New York Academy of Sciences. (1996) 791, no. 1, 369–377, 10.1111/j.1749-6632.1996.tb53544.x, 2-s2.0-0029736838. 8784518

[87] Tealdo M. , Romero G. , Autrey C. , and Samartino L. , Serología Positiva a *Leptospira interrogans*, Serovar Cynopteri en Caninos de la Ciudad de Buenos Aires, Argentina, InVet. (2007) 9, no. 1, 59–65.

[88] de Oliveira D. , Khalil H. , Palma F. A. G. , Santana R. , Nery N. , and Quintero-Vélez J. C. , Factors Associated with Differential Seropositivity to *Leptospira interrogans* and *Leptospira kirschneri* in a High Transmission Urban Setting for Leptospirosis in Brazil. medRxiv, 2023. 10.1371/journal.pntd.0011292 PMC11139309 38758957

[89] Murillo A. , Cuenca R. , and Serrano E. , et al.Leptospira Detection in Cats in Spain by Serology and Molecular Techniques, International Journal of Environmental Research and Public Health. (2020) 17, no. 5, 10.3390/ijerph17051600, 1600. 32121670 PMC7084519

[90] Parreira I. , Jayme V. , Buzin E. , Tomaz L. , and Delfino D. A. , Epidemiological Features of Infection Through Leptospira Spp. in Domestic Cats (*Felis catus*) Apparently Healthy Within the Metropolitan Area of Goiânia, Brazil, Enciclopédia Biosfera. (2010) 6, 9–11.

[91] Chatfield J. , Milleson M. , Stoddard R. , Bui D. M. , and Galloway R. , Serosurvey of Leptospirosis in Feral Hogs (*Sus scrofa*) in Florida, Journal of Zoo and Wildlife Medicine. (2013) 44, no. 2, 404–407, 10.1638/2012-0258R2.1, 2-s2.0-84878924636. 23805559

[92] Levett P. N. , Leptospirosis, Clinical Microbiology Reviews. (2001) 14, no. 2, 296–326, 10.1128/CMR.14.2.296-326.2001, 2-s2.0-0035072803. 11292640 PMC88975

[93] Nally J. E. , Ahmed A. A. A. , Putz E. J. , Palmquist D. E. , and Goris M. G. A. , Comparison of Real-Time PCR, Bacteriologic Culture and Fluorescent Antibody Test for the Detection of *Leptospira borgpetersenii* in Urine of Naturally Infected Cattle, Veterinary Sciences. (2020) 7, no. 2, 10.3390/vetsci7020066, 66. 32429076 PMC7356886

[94] Rinehart C. L. , Zimmerman A. D. , Buterbaugh R. E. , Jolie R. A. , and Chase C. C. , Efficacy of Vaccination of Cattle With the *Leptospira interrogans* Serovar Hardjo Type Hardjoprajitno Component of a Pentavalent Leptospira Bacterin Against Experimental Challenge With *Leptospira borgpetersenii* Serovar Hardjo Type Hardjo-Bovis, American Journal of Veterinary Research. (2012) 73, no. 5, 735–740, 10.2460/ajvr.73.5.735, 2-s2.0-84860317995. 22533408

[95] Bolin C. , Thiermann A. , Handsaker A. , and Foley J. , Effect of Vaccination With a Pentavalent Leptospiral Vaccine on *Leptospira interrogans* Serovar Hardjo Type Hardjo-Bovis Infection of Pregnant Cattle, American Journal of Veterinary Research. (1989) 50, no. 1, 161–165, 10.2460/ajvr.1989.50.01.161. 2645816

[96] Steven E. W. , Rogers G. M. , and Ramachandran S. , et al.Herd Prevalence and Risk Factors of Leptospira Infection in Beef Cow/Calf Operations in the United States: *Leptospira borgpetersenii* Serovar Hardjo, The Bovine Practitioner. (2007) 15–23.

[97] Chideroli R. , Pereira U. , and Gonçalves D. , et al.Isolation and Molecular Characterization of *Leptospira borgpetersenii* Serovar Hardjo Strain Hardjobovis in the Urine of Naturally Infected Cattle in Brazil, Genetics and Molecular Research. (2016) 15, no. 1, 10.4238/gmr.15018473, 2-s2.0-84961755651, 19. 26909976

[98] Guedes I. B. , de Almeida Araújo S. A. , and de Souza G. O. , et al.Circulating Leptospira Species Identified in Cattle of the Brazilian Amazon, Acta Tropica. (2019) 191, 212–216, 10.1016/j.actatropica.2019.01.011, 2-s2.0-85059893730. 30639452

[99] Hamond C. , Pinna M. , Medeiros M. A. , Bourhy P. , Lilenbaum W. , and Picardeau M. , A Multilocus Variable Number Tandem Repeat Analysis Assay Provides High Discrimination for Genotyping *Leptospira santarosai* Strains, Journal of Medical Microbiology. (2015) 64, no. 5, 507–512, 10.1099/jmm.0.000045, 2-s2.0-84930971457. 25721051 PMC4857445

[100] Guedes I. B. , de Souza G. O. , and de Souza Rocha K. , et al.Leptospira Strains Isolated From Cattle in the Amazon Region, Brazil, Evidence of a Variety of Species and Serogroups With a High Frequency of the Sejroe Serogroup, Comparative Immunology, Microbiology and Infectious Diseases. (2021) 74, 10.1016/j.cimid.2020.101579, 101579. 33246243

[101] Aymée A. L. , Di Azevedo M. I. N. , de Souza Pedrosa J. , d. S. L. de Melo J. , Carvalho-Costa F. A. , and Lilenbaum W. , The Role of *Leptospira santarosai* Serovar Guaricura as Agent of Bovine Genital Leptospirosis, Veterinary Microbiology. (2022) 268, 10.1016/j.vetmic.2022.109413, 109413. 35390628

[102] Loureiro A. P. , Hamond C. , Pinto P. , Bremont S. , Bourhy P. , and Lilenbaum W. , Molecular Analysis of Leptospires From Serogroup Sejroe Obtained From Asymptomatic Cattle in Rio de Janeiro—Brazil Reveals Genetic Proximity to Serovar Guaricura, Research in Veterinary Science. (2016) 105, 249–253, 10.1016/j.rvsc.2016.02.012, 2-s2.0-84960453646. 27033941

[103] Rivera P. , Ticlla M. , Balda L. , Gonzalez D. , and Céspedes M. , Diversidad Genética de Aislamientos Peruanos de Leptospira spp. Mediante Electroforesis en gel de Campo Pulsado, Revista Peruana de Medicina Experimental y Salud Pública. (2012) 29, no. 4, 469–476, 10.1590/S1726-46342012000400008, 2-s2.0-84873966665. 23338631

[104] Carmona-Gasca C. A. , Lara L. L. , Castillo-Sánchez L. O. , Ramírez-Ortega J. M. , Palomera C. L. , and de la Peña-Moctezuma A. , Detection of *Leptospira santarosai* and *L. kirschneri* in Cattle: New Isolates With Potential Impact in Bovine Production and Public Health, Veterinaria Mexico. (2011) 42, no. 4, 277–288.

[105] Chiebao D. P. , Valadas S. Y. O. B. , and Minervino A. H. H. , et al.Variables Associated With Infections of Cattle by *Brucella abortus*., *Leptospira spp*. and *Neospora spp*. in Amazon Region in Brazil, Transboundary and Emerging Diseases. (2015) 62, no. 5, e30–e36, 10.1111/tbed.12201, 2-s2.0-84940479372. 26302373

[106] Cosate M. R. V. , Sakamoto T. , and de Oliveira Mendes T. A. , et al.Molecular Typing of *Leptospira interrogans* Serovar Hardjo Isolates From Leptospirosis Outbreaks in Brazilian Livestock, BMC Veterinary Research. (2017) 13, no. 1, 1–12, 10.1186/s12917-017-1081-9, 2-s2.0-85027569097. 28619055 PMC5471881

[107] Govindan B. , Involvement of Leptospira Serovars With Different Clinical Conditions of Leptospirosis in Cattle, Biomedical & Pharmacology Journal. (2014) 7, no. 1, 125–128, 10.13005/bpj/461, 2-s2.0-84908118768.

[108] Thurmond M. C. , Picanso J. P. , and Hietala S. K. , Prospective Serology and Analysis in Diagnosis of Dairy Cow Abortion, Journal of Veterinary Diagnostic Investigation. (1990) 2, no. 4, 274–282, 10.1177/104063879000200404, 2-s2.0-0025502337. 1965632

[109] Black P. , Corney B. , Smythe L. , Dohnt M. , Norris M. , and Symonds M. , Prevalence of Antibodies to Leptospira Serovars in Beef Cattle in Central Queensland, Australian Veterinary Journal. (2001) 79, no. 5, 344–348, 10.1111/j.1751-0813.2001.tb12010.x, 2-s2.0-0035345975. 11432001

[110] Nervig R. , Beran C. , and Hill H. , Bovine Leptospirosis in Iowa: A Serological Survey, 1980.

[111] Lilenbaum W. and Martins G. , Leptospirosis in Cattle: A Challenging Scenario for the Understanding of the Epidemiology, Transboundary and Emerging Diseases. (2014) 61, 63–68, 10.1111/tbed.12233, 2-s2.0-84906076079. 25135465

