# Supplementary material for: Leptospira Species Infection and Seropositivity in Domestic Livestock and Feral Swine in Puerto Rico
Source: Transbound Emerg Dis. 2026 May 27;2026:2538118. doi: 10.1155/tbed/2538118 (PMC13213916; doi:10.1155/tbed/2538118)
Supplement: Supplementary file 1 — Supporting Information 1 Table S1. ZSAL and NCAH microscopic agglutination test antigen panels for Leptospira serogroup testing of domestic animal and feral swine samples collected in Puerto Rico from August 2019 to September 2021. Figure S1. Leptospirosis outreach document provided to owners of animals involved in the investigation of leptospirosis in domestic livestock and feral swine in Puerto Rico from August 2019 to September 2021 (English and Spanish versions provided). [file TBED-2026-2538118-s004.pdf]

**Supplementary Table 1.** USDA National Center for Animal Health (NCAH) and Centers for Disease Control and Prevention Zoonoses and Select Agent Laboratory (ZSAL) microscopic agglutination test (MAT) antigen panels for *Leptospira* serogroup testing of domestic animal and feral swine samples collected in Puerto Rico from August 2019-September 2021

| **Serogroup** | **Serovar** | **MAT Antigen Panel NCAH (Panel A)** | **MAT Antigen Panel ZSAL (Panel B)** |
|---|---|---|---|
| Australis | Australis | X | X |
| Australis | Bratislava | X | X |
| Autumnalis | Autumnalis | X | X |
| Ballum | Ballum | X | X |
| Bataviae | Bataviae | X | X |
| Canicola | Canicola | X | X |
| Celledoni | Celledoni | | X |
| Cynopteri | Cynopteri | X | X |
| Djasiman | Djasiman | X | X |
| Grippotyphosa | Grippotyphosa | X | X |
| Hebdomadis | Borincana | | X |
| Hebdomadis | Hebdomadis | X | |
| Icterohaemorrhagiae | Copenhageni | X | |
| Icterohaemorrhagiae | Icterohaemorrhagiae | | X |
| Icterohaemorrhagiae | Mankarso | | X |
| Javanica | Javanica | | X |
| Mini | Georgia | | X |
| Mini | Szwajizak | X | |
| Pomona | Pomona | X | X |
| Pyrogenes | Alexi | | X |
| Pyrogenes | Pyrogenes | X | X |
| Sejroe | Hardjo | X | |
| Sejroe | Recreo | X | |
| Sejroe | Sejroe | X | |
| Sejroe | Wolffi | | X |
| Tarassovi | Tarassovi | X | X |
| ND | Room 22 | X | |

DRAFT

**Supplementary Figure 1.** Leptospirosis outreach document provided to owners of animals involved in the investigation of leptospirosis in domestic livestock and feral swine in Puerto Rico from August 2019 – September 2021 (English and Spanish versions provided).

# LEPTOSPIROSIS:
## Protect Yourself While Working with Farm Animals that May Have Leptospirosis

### What is leptospirosis?

Leptospirosis is a bacterial disease that can cause serious illness in animals and people. People working with livestock and other animals are at risk of getting the disease.

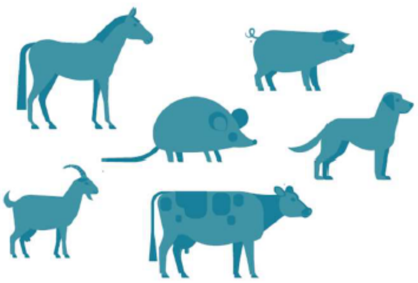

Some types animals that can carry leptospirosis

### How do people get leptospirosis?

**People can get leptospirosis when they come in contact with:**

- urine from infected animals, which can include cows, pigs, sheep, goats, horses, rodents, dogs, and wildlife
- reproductive tissues and fluids from infected animals (like the placenta and amniotic fluid)
- freshwater contaminated with urine of infected animals (like rivers or streams, lakes, floodwater, collected rainwater, or wet soil)
- urine-contaminated food or water (by eating or drinking)

The bacteria can enter the body through cuts or scratches or through the eyes, nose, or mouth. The risk of infection for animals and people can increase after heavy rains or flooding.

### What are the symptoms?

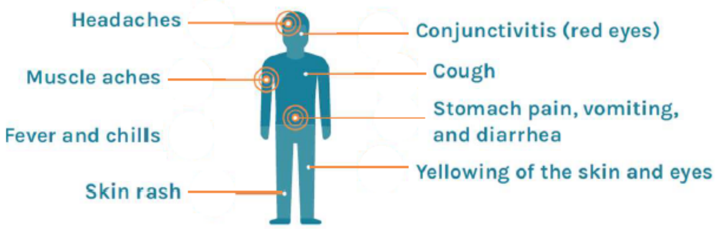


### What should I do if I'm sick or have been exposed?

- If you develop symptoms of leptospirosis, see a doctor as soon as possible. Tell your doctor if you have had any potential exposures to leptospirosis.
- If you are exposed to an animal with leptospirosis, monitor yourself closely for symptoms for 30 days after your last exposure.
- If you are exposed to an animal with leptospirosis and are immunocompromised or pregnant, talk to a doctor even if you do not have symptoms.
- If you have leptospirosis, early treatment with antibiotics may help prevent more severe illness and shorten the time you are sick. Without treatment, leptospirosis illness can get worse—people can develop kidney and liver failure, meningitis, difficulty breathing, bleeding—and, in some cases, people may die from their infection.

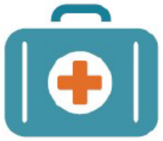

## How can I protect myself?

- Wear waterproof protective clothing, boots or closed shoes, and gloves when working with animals that may have leptospirosis, animal waste, or water or soil that could be contaminated with animal urine.
- Do not eat, drink, or smoke while working with or near animals or animal waste.
- Wash hands with soap and water after working with animals or animal waste.
- Cover cuts or scratches with waterproof bandages or other coverings when working with animals or animal waste.
- During activities with a high risk of being splashed by urine, reproductive fluids, or other body fluids (like hosing down animal areas, milking, or assisting a birth or abortion), wear eye and face protection such as safety goggles and surgical mask or a face shield.
- Avoid activities that may create mist in the air from urine or urine-contaminated water, such as high-pressure hosing when cleaning animal areas or waste products.
- Keep rodents away by keeping garbage and animal feed in secure containers and trapping rodents. Wear gloves if handling materials that may be contaminated with rodent urine or excrement. Prevent wildlife access to livestock and animal barns.
- Don't wade in, swim in, or drink fresh water that may be contaminated with animal urine or floodwater (like in a river, trench, or pond), especially fresh water near animals or with potential runoff from animal areas. Also avoid using untreated water sources as water supply for animals.
- Treat potentially contaminated water to make it safe for drinking by boiling or chemically treating.
- Consider vaccinating pets and livestock for leptospirosis.

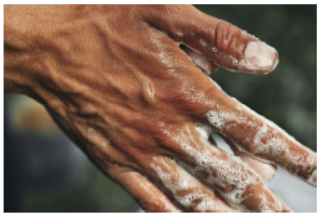
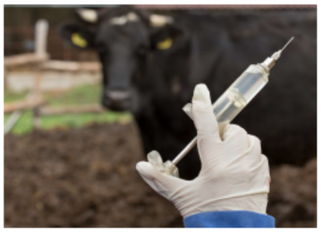
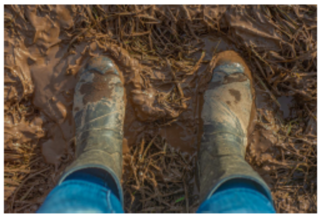
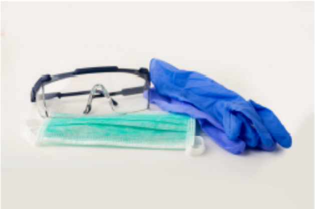
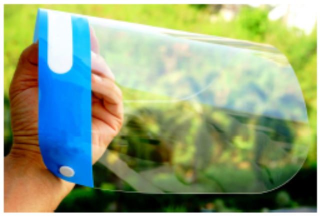
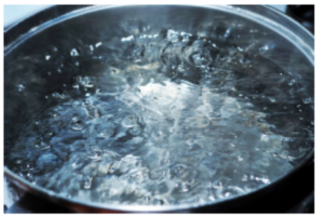

**For more information from CDC, visit www.cdc.gov/leptospirosis**
