# Supplementary material for: Leptospira Species Infection and Seropositivity in Domestic Livestock and Feral Swine in Puerto Rico
Source: Transbound Emerg Dis. 2026 May 27;2026:2538118. doi: 10.1155/tbed/2538118 (PMC13213916; doi:10.1155/tbed/2538118)
Supplement: Supplementary file 4 — Supporting Information 4 File S3. secY PCR sequencing results for dairy cattle urine samples collected in Puerto Rico during the investigation of leptospirosis in domestic livestock and feral swine from August 2019 to September 2021. [file TBED-2026-2538118-s003.pdf]

secY sequences

DCP-008 (clinical sample – urine)

ATGCCGATCATTTTTGCTTCTTCCTTGATCCTGTTTCCACAGACGATCATTCAGTGGTTGTCTTCCAGTA
GCGAACAATGGGCGGGTTGGGCGATCATTATGGACTTTTTCAATCCATTCTCCCAGATCTGGTATCATGC
ATTGTTCTACTATATAATCTATACTTCTTTGATCGTGTTCTTCGCATACTTTTATACTGCGATTCAGTTC
AACCCTGCAGAGTTGGCGGAAAACCTGAAAAAATACGGCGGATTCATTCCAGGAATTCGTCCCGGTTCTC
ATACGAAAGAATACATCGAAAAAGTGTTAAACAGAATCACTCTCCCCGGTGCGATGTTCCTCGCGGGATT
GGCCCTGGCTCCTTACATCATCATCAAATTCTTAGATTTGAGTTCCAACTCAGGCGGCGGGTCCCTGGTT
TATACTTTTGGTGGGACGTCTCTCTTGATCATGGTAGGGGTTGCGCTCGAGACTCTGAAACAGATCGAGT
CTCAACTTTTGATGAGAAATTATGAAGGCTTCATGAAGAAGTCTAAAATTAAGGGACGG

DCP-009 (culture)

ATGCCGATCATTTTTGCTTCTTCCTTGATCCTGTTTCCACAGACGATCATTCAGTGGTTGTCTTCCAGTA
GCGAACAATGGGCGGGTTGGGCGATCATTATGGACTTTTTCAATCCATTCTCCCAGATCTGGTATCATGC
ATTGTTCTACTATATAATCTATACTTCTTTGATCGTGTTCTTCGCATACTTTTATACTGCGATTCAGTTC
AACCCTGCAGAGTTGGCGGAAAACCTGAAAAAATACGGCGGATTCATTCCAGGAATTCGTCCCGGTTCTC
ATACGAAAGAATACATCGAAAAAGTGTTAAACAGAATCACTCTCCCCGGTGCGATGTTCCTCGCGGGATT
GGCCCTGGCTCCTTACATCATCATCAAATTCTTAGATTTGAGTTCCAACTCAGGCGGCGGGTCCCTGGTT
TATACTTTTGGTGGGACGTCTCTCTTGATCATGGTAGGGGTTGCGCTCGAGACTCTGAAACAGATCGAGT
CTCAACTTTTGATGAGAAATTATGAAGGCTTCATGAAGAAGTCTAAAATTAAGGGACGG

DCP-011 (clinical sample – urine)

ATGCCGATCATTTTTGCTTCTTCCTTGATCCTGTTTCCACAGACGATCATTCAGTGGTTGTCTTCCAGTA
GCGAACAATGGGCGGGTTGGGCGATCATTATGGACTTTTTCAATCCATTCTCCCAGATCTGGTATCATGC
ATTGTTCTACTATATAATCTATACTTCTTTGATCGTGTTCTTCGCATACTTTTATACTGCGATTCAGTTC
AACCCTGCAGAGTTGGCGGAAAACCTGAAAAAATACGGCGGATTCATTCCAGGAATTCGTCCCGGTTCTC
ATACGAAAGAATACATCGAAAAAGTGTTAAACAGAATCACTCTCCCCGGTGCGATGTTCCTCGCGGGATT
GGCCCTGGCTCCTTACATCATCATCAAATTCTTAGATTTGAGTTCCAACTCAGGCGGCGGGTCCCTGGTT
TATACTTTTGGTGGGACGTCTCTCTTGATCATGGTAGGGGTTGCGCTCGAGACTCTGAAACAGATCGAGT
CTCAACTTTTGATGAGAAATTATGAAGGCTTCATGAAGAAGTCTAAAATTAAGGGACGG

DCP-017 (culture)

ATGCCGATCATTTTTGCTTCTTCCTTGATTCTGTTCCCACAGACGATCATTCAGTGGTTGTCTTCCAGCA
GCGAACAGTGGGCGGGTTGGGCGATCATTATGGACTTTTTCAATCCGTTCTCCCAGATCTGGTATCACGC
ATTGTTCTACTTTGTGATCTATACTTCTTTGATTATTTTCTTCGCATACTTTTACACTGCGATTCAGTTC
AACCCTGCGGAGTTGTCCGAAAACCTGAAGAAATACGGCGGGTTCATTCCAGGTATTCGTCCCGGTTCTC
ACACAAAAGAATACATCGAAAAGGTGTTAAACAGAATCACACTTCCCGGCGCGATGTTCCTCGCGGGATT
GGCTCTGGCTCCTTACATCATCATCAAATTCTTAGATTTGAGCTCCAACTCTGGAGGAGGATCTCTGGTT
TATACCTTCGGCGGAACGTCCCTCTTGATTATGGTAGGGGTTGCGCTCGAGACTTTGAAACAAATCGAGT
CTCAACTTTTGATGAGAAACTACGAAGGCTTCATGAAGAAGTCTAAAATTAAGGGACGG

DCP-041 (culture)

ATGCCGATCATTTTTGCTTCTTCCTTGATCCTGTTTCCACAGACGATCATTCAGTGGTTGTCTTCCAGTA
GCGAACAATGGGCGGGTTGGGCGATCATTATGGACTTTTTCAATCCATTCTCCCAGATCTGGTATCATGC
ATTGTTCTACTATATAATCTATACTTCTTTGATCGTGTTCTTCGCATACTTTTATACTGCGATTCAGTTC
AACCCTGCAGAGTTGGCGGAAAACCTGAAAAAATACGGCGGATTCATTCCAGGAATTCGTCCCGGTTCTC
ATACGAAAGAATACATCGAAAAAGTGTTAAACAGAATCACTCTCCCCGGTGCGATGTTCCTCGCGGGATT

GGCCCTGGCTCCTTACATCATCATCAAATTCTTAGATTTGAGTTCCAACTCAGGCGGCGGGTCCCTGGTT
TATACTTTTGGTGGGACGTCTCTCTTGATCATGGTAGGGGTTGCGCTCGAGACTCTGAAACAGATCGAGT
CTCAACTTTTGATGAGAAATTATGAAGGCTTCATGAAGAAGTCTAAAATTAAGGGACGG

BCP-006, DCP-022, DCP-073, DCP-084: negative by *secY*
